# *Momordica charantia* L. Confers Multifaceted Protection Against 5-Fluorouracil-Induced Intestinal Injury via Inhibition of Inflammation, Oxidative Stress, Epithelial-Mesenchymal Transition, and Tight Junction Disruption

**DOI:** 10.3390/antiox15040431

**Published:** 2026-03-30

**Authors:** Wen-Ping Jiang, Jaung-Geng Lin, Atsushi Inose, Wen-Liang Wu, Song-Jie Chiang, Guan-Jhong Huang

**Affiliations:** 1 School of Pharmacy, China Medical University, Taichung 404, Taiwan; wpjiang@cmu.edu.tw; 2 School of Chinese Medicine, College of Chinese Medicine, China Medical University, Taichung 404, Taiwan; jglin@mail.cmu.edu.tw (J.-G.L.);; 3 Chinese Medicine Research Center, China Medical University, Taichung 404, Taiwan; 4 Faculty of Pharmacy, Nihon Pharmaceutical University, Ina-machi Komuro, Kita-Adachi-gun, Saitama 362-0806, Japan; ainose@nichiyaku.ac.jp; 5 Department of Chinese Pharmaceutical Sciences and Chinese Medicine Resources, College of Chinese Medicine, China Medical University, Taichung 404, Taiwan; jj22135743@gmail.com; 6 Department of Food Nutrition and Health Biotechnology, Asia University, Taichung 413, Taiwan

**Keywords:** *Momordica charantia* L., 5-fluorouracil, mucositis, oxidative stress, inflammation, apoptosis, epithelial–mesenchymal transition, tight junction

## Abstract

*Momordica charantia* L. (MC), also referred to as bitter gourd or bitter melon, is a Cucurbi taceae plant renowned for its medicinal benefits. 5-Fluorouracil (5-FU) is employed as a frontline chemotherapeutic agent, with its antitumor activity mediated through the inhibition of DNA and RNA synthesis. However, its therapeutic efficacy is often compromised by serious adverse effects, particularly gastrointestinal inflammation. Therefore, this research examined the efficacy of the ethanolic extract of *Momordica charantia* fruit (EMC) in mitigating 5-FU-induced intestinal mucositis in mice. Mucositis was induced in mice by intraperitoneal administration of 5-FU at 50 mg/kg from experimental days 4 to 7, with EMC administered orally at doses of 125 mg/kg and 250 mg/kg once daily for ten consecutive days. 5-FU exposure resulted in severe intestinal injury, manifested by markedly upregulated inflammation and oxidative stress. EMC treatment significantly reversed these pathophysiological alterations, restoring mucosal architecture and function. Furthermore, EMC effectively reduced the 5-FU-induced release of inflammatory mediators and oxidative stress markers. These results demonstrate that EMC acts as a novel protective modulator of 5-FU-induced mucositis, offering substantial translational potential as an adjunctive supportive therapy in colorectal cancer management.

## 1. Introduction

Chemotherapy, alongside radiotherapy and surgery, remains a cornerstone of cancer management. Its prominence stems from a proven ability to prolong survival and reduce tumor burden [1]. However, this therapeutic benefit is frequently offset by a fundamental lack of specificity [2]. Cytotoxic agents inevitably affect not only rapidly dividing cancer cells but also healthy tissues with high proliferative capacity, such as the gastrointestinal mucosa, bone marrow, and hair follicles. The systemic administration of these agents disrupts cellular homeostasis, induces oxidative stress, and depletes endogenous antioxidants, collectively leading to impaired immune function and severe clinical side effects—including mucositis, myelosuppression, and heightened infection risk [3]. These adverse outcomes underscore the critical need for adjunctive strategies that can mitigate treatment-related toxicity without compromising anticancer efficacy.

The clinical efficacy of 5-fluorouracil (5-FU) against solid tumors is often offset by its significant off-target toxicity. Its mechanism as an antimetabolite, while effective at disrupting nucleic acid synthesis in cancer cells, also damages healthy proliferating tissues [4]. The intestinal mucosa, with its high cellular turnover, is particularly susceptible, frequently leading to dose-limiting mucositis. This condition manifests as barrier disruption, villous atrophy, and inflammation, clinically presenting as diarrhea and pain in 50–80% of patients [5]. Such adverse effects not only diminish quality of life but also necessitate treatment modifications that can compromise therapeutic success [6]. Currently, management strategies are primarily palliative and provide only partial relief without addressing the underlying pathophysiology of epithelial injury and oxidative stress [7]. Therefore, the development of adjunctive therapies—particularly bioactive compounds with antioxidant and anti-inflammatory properties—represents a promising strategy to mitigate 5-FU-induced toxicity while preserving its anticancer efficacy.

Mucositis, a common and dose-limiting complication of chemotherapy, is a complex pathophysiological process that evolves through distinct yet overlapping phases: initiation, primary damage response, signal amplification, ulceration, and finally, healing [8]. The process is initiated by direct cellular insult—including DNA strand breaks and the accumulation of reactive oxygen species (ROS)—within the rapidly dividing epithelial cells of the mucosa. This initial oxidative damage activates key signaling cascades, most notably the nuclear factor-κB (NF-κB) pathway, leading to the upregulation of pro-inflammatory cytokines [e.g., tumor necrosis factor-α (TNF-α), interleukin-1β (IL-1β), interleukin-6 (IL-6)] and tissue-degrading enzymes [9]. During the amplification phase, this local inflammatory response intensifies, culminating in widespread mucosal barrier breakdown, ulcer formation, severe pain, and secondary microbial invasion, which heightens the risk of systemic infection. Healing is a dynamic but often impaired process [3]. Cytotoxic chemotherapy directly interferes with epithelial regeneration by disrupting DNA synthesis and inhibiting critical pathways, such as thymidylate synthesis, which are essential for cellular replication [10]. Concurrently, persistent ROS generation induces mitochondrial dysfunction and apoptotic cascades within epithelial cells. The resulting oxidative stress not only compromises cell survival but also perpetuates inflammatory signaling via pathways like NF-κB, creating a self-sustaining cycle of injury. This vicious interplay between oxidative damage and chronic inflammation significantly delays mucosal restitution [11]. Thus, the pathology of mucositis is characterized by a synergistic loop of oxidative stress and inflammatory activation. Effective therapeutic intervention, therefore, necessitates a dual strategy that concurrently targets both pathways to disrupt this cycle and promote timely tissue repair.

Epithelial–mesenchymal transition (EMT) serves as a pivotal driver of tumor progression, invasion, and metastasis, significantly contributing to poor prognosis and therapeutic resistance [4]. In the context of 5-FU-induced intestinal mucositis, EMT plays a critical role in exacerbating mucosal injury. EMT exacerbates this loss of barrier integrity by promoting the conversion of intestinal epithelial cells from a polarized, adhesive epithelial phenotype to a mesenchymal phenotype characterized by enhanced migratory and invasive capacities [12]. Furthermore, key mediators such as TNF-α and oxidative stress exacerbate epithelial permeability by altering the expression, phosphorylation, and cellular localization of tight junction components. The breakdown of tight junctions, which is essential for maintaining the selective barrier function of the intestinal epithelium, has been well-documented in preclinical models of 5-FU-induced mucositis. These junctions strictly regulate paracellular flux to prevent the translocation of pathogens and toxins [13]. Despite these insights, the precise molecular alterations and regulatory mechanisms governing EMT and tight junction dynamics during chemotherapy-induced injury remain insufficiently characterized, highlighting a significant gap in the current understanding of mucositis pathophysiology.

*Momordica charantia* L. (MC)—widely known as bitter melon, balsam pear, or karela—is a member of the Cucurbitaceae family distinguished by its extensive morphological and genetic heterogeneity [14]. These differences span multiple biological dimensions, including reproductive traits, vegetative morphology, and fruit ontogeny. MC has gained considerable attention in both traditional and contemporary therapeutic systems because of its diverse pharmacological activities, including antioxidant, anti-inflammatory, antitumor, hypoglycemic, and immunomodulatory effects, which collectively support systemic homeostasis and organ protection. MC has been widely recognized as a functional dietary component due to its bioactive constituents, including charantin, vicine, polypeptide-p, mormordin, carotenoids, quercetin, and gallic acid [15]. The ethanolic extract of *Momordica charantia* fruit (EMC) is particularly recognized for its glucose-lowering capacity with a favorable safety profile [16]. EMC and its bioactive constituents (p-coumaric acid, rutin, and quercetin) may disrupt severe acute respiratory syndrome coronavirus 2 (SARS-CoV-2) entry into host cells by modulating the Angiotensin-converting enzyme 2 (ACE2)/Transmembrane protease, serine 2 (TMPRSS2) axis [17]. This study aimed to evaluate the ameliorative efficacy of EMC against 5-FU-induced intestinal mucositis and to elucidate the molecular pathways contributing to its protective mechanism.

## 2. Materials and Methods

### 2.1. Materials

The fruit of *M. charantia* was harvested in 2024 from Taichung, Taiwan, and taxonomically identified by Prof. Guan-Jhong Huang of China Medical University. A voucher specimen was deposited in the Department of Chinese Pharmaceutical Sciences and Chinese Medicine Resources at China Medical University (Accession No. CMU-202401). Bitter melon fruits (1 kg) were air-dried in the shade, ground into a fine powder, and subjected to extraction using 95% ethanol at a 1:4 (*w*/*v*) ratio. The extraction process was carried out at 65 °C for 2 h. The resulting extract was filtered twice through muslin cloth and Whatman filter paper (100 mm), concentrated under reduced pressure to a viscous residue, freeze-dried for three days, and finally stored at −20 °C until use.

### 2.2. Reagents

All chemicals, including 5-FU and mesalazine, were obtained from Sigma-Aldrich (St. Louis, MO, USA). Primary antibodies used in this study were obtained from the following commercial sources. Antibodies against inducible nitric oxide synthase (iNOS; GTX31048), cyclooxygenase-2 (COX-2; GTX100656), inhibitor of kappa B alpha (IκBα; GTX110521), phosphorylated-IκBα (p-IκBα; GTX25682), nuclear factor kappa B (NF-κB; GTX107678), phosphorylated-NF-κB (p-NF-κB; GTX50098), phosphorylated-ERK (p-ERK; GTX59568), phosphorylated-JNK (p-JNK; GTX50868), catalase (GTX110704), glutathione peroxidase 3 (GPx3; GTX89142), heme oxygenase-1 (HO-1; GTX101147), B-cell lymphoma 2 (Bcl-2; GTX100064), Bcl-2-associated X protein (Bax; GTX109683), epithelial cadherin (E-cadherin; GTX100443), neural cadherin (N-cadherin; GTX112734), occludin (GTX114949), claudin-1 (GTX134842), and zonula occludens-1 (ZO-1; GTX636491) were purchased from GeneTex, Inc. (Irvine, CA, USA). All were used at a 1:1000 dilution. Antibodies for Toll-like receptor 4 (TLR4; A5258), nuclear factor erythroid 2-related factor 2 (Nrf2; A1244), and cysteine-aspartic protease 3 (caspase-3; A16793) were obtained from Abclonal Technology, Inc. (Woburn, MA, USA) at a 1:1000 dilution. Antibodies against IκB kinase (IKK; 2678), phosphorylated-IKK (p-IKK; 2078), extracellular signal-regulated kinase (ERK; 4695S), and phosphorylated-p38 (p-p38; 4631S) were sourced from Cell Signaling Technology, Inc. (Danvers, MA, USA) and used at a 1:1000 dilution. Antibodies against p38 (ab31828), β-catenin (ab6302), glycogen synthase kinase-3 beta (GSK-3β; ab18893), phosphorylated-GSK-3β (p-GSK-3β; ab279814), and cyclin D1 (ab24249) were sourced from Abcam (Cambridge, UK). Antibodies for c-jun N-terminal kinase (JNK; 06-748) and superoxide dismutase 1 (SOD1; 70658) were obtained from MilliporeSigma (Burlington, MA, USA) and BioVision, Inc. (Milpitas, CA, USA), respectively. All primary antibodies were used at a 1:1000 dilution. Finally, an antibody against β-Actin (R380624; 1:2000 dilution) was purchased from Zen-BioScience (Chengdu, China) and served as a loading control [18].

### 2.3. Animals

Male BALB/c mice (8 weeks old, 25 ± 3 g; BioLASCO Taiwan Co., Ltd., Taipei, Taiwan) were housed under controlled conditions (12 h light/dark cycle, 23 °C, 50% humidity) and acclimated for 3–5 days before the experiments. All study protocols were conducted in accordance with the Institutional Guidelines of the China Medical University for the Care and Use of Experimental Animals (IGC-MU-CUEA) and were approved by the Institutional Animal Care and Use Committee of the China Medical University (IACUC-CMU; Taichung, Taiwan; Protocol no. CMUIACUC-2022-481). All procedures were performed in accordance with the institutional guidelines for the care and use of laboratory animals, which are established based on and fully consistent with internationally accepted principles, including the ‘3Rs’ (Replacement, Reduction, and Refinement).

### 2.4. Research Design

A total of twenty-five male BALB/c mice were utilized in this study and randomly assigned into five experimental groups (n = 5 per group): (1) a saline-treated control group; (2) a 5-fluorouracil (5-FU)-treated group (50 mg/kg, i.p.); (3) a mesalazine-treated group (10 mg/kg, i.p.) combined with 5-FU (50 mg/kg, i.p.); (4) a low-dose EMC group (125 mg/kg, orally) co-administered with 5-FU (50 mg/kg, i.p.); and (5) a high-dose EMC group (250 mg/kg, orally) co-administered with 5-FU (50 mg/kg, i.p.). Given its established clinical efficacy in treating inflammatory bowel diseases, mesalazine (5-aminosalicylic acid, 5-ASA) has been adopted as a reference compound in preclinical models of intestinal injury. In the context of 5-FU-induced mucositis, it reliably attenuates diarrhea, weight loss, and mucosal inflammation, primarily through the inhibition of pro-inflammatory mediators and preservation of tissue integrity.

Mice were allocated into control, 5-FU, and treatment groups. The control and 5-FU groups received physiological saline by oral gavage from day 1 to day 10, whereas other groups received their respective treatment regimens. To establish the mucositis model, 5-FU (50 mg/kg) was administered intraperitoneally from days 4 to 7 in all groups except the control. On the final experimental day, following a 16 h fast, mice were euthanized by overdose of 5% isoflurane. The complete small and large intestines were immediately excised, gently flushed with ice-cold 0.9% saline, and measured for length. Specimens were subsequently processed for histopathological examination and for Western blot analysis of target proteins, including inflammatory mediators, components of the MAPK signaling pathway, apoptosis-related proteins, antioxidant enzymes, and tight junction regulators. Daily monitoring of body weight and diarrhea indices served as functional indicators of mucosal integrity and therapeutic efficacy. A representative segment of intestinal tissue from each group was fixed in 10% phosphate-buffered formalin for subsequent histopathological evaluation. Terminal blood samples were obtained by cardiac puncture, centrifuged at 1100× *g* for 10 min to isolate serum, and stored at −80 °C until biochemical analysis [18].

### 2.5. Daily Weight and Diarrhea Monitoring

Body weight was measured on days 0, 3, 5, 8, and 10. Diarrhea severity was quantified on day 10 by averaging the scores obtained. The severity of diarrhea was graded using Bowen’s criteria: grade 0 indicated normal pellets; grade 1 (mild) corresponded to slightly wet and soft stools with staining limited to the perianal fur; grade 2 (moderate) was assigned upon observation of loose stools with staining extending to the hind legs; and grade 3 (severe) was reserved for cases presenting with watery stools (with or without mucous), extensive staining covering the hind legs and abdomen, and frequently, continuous anal oozing. For each experimental group, the overall diarrheal severity was expressed as a composite measure integrating incidence rate and mean severity score [18].

### 2.6. Histological Examination

Intestinal tissues were fixed in formalin, embedded in paraffin, and sectioned at 5 µm. Sections were stained with H&E and examined morphologically using a Nikon Eclipse TS100 microscope. Lesion severity was graded as follows: 0 = normal, 1 < 25%, 2 = 25–50%, 3 = 50–75%, 4 > 75%. For Ki-67 detection, slides were deparaffinized, rehydrated, and incubated with a Ki-67 primary antibody (4 °C, 24 h). Immunoreactivity was visualized with DAB substrate (Sigma-Aldrich, St. Louis, MO, USA) [19].

### 2.7. Cytokine Assay

Quantitative assessment of serum TNF-α, IL-1β, and IL-6 (cat. No. 430904, 432601, and 431307) concentrations was conducted using enzyme-linked immunosorbent assay (ELISA) kits (BioLegend, San Diego, CA, USA). All steps were executed in strict adherence to the supplier’s technical guidelines and protocol specifications. Standard curves were generated using the provided cytokine standards, and sample concentrations were calculated by interpolation from the respective standard curves to ensure high sensitivity, accuracy, and reproducibility of the measurements [18].

### 2.8. Nitrite Assay

Nitrite concentrations in serum were quantitatively assessed using the Griess colorimetric assay, which relies on the diazotization of sulfanilamide and coupling with *N*-(1-naphthyl) ethylenediamine to produce a measurable azo dye. In brief, 100 μL of Griess reagent was added to each sample aliquot, followed by gentle agitation and incubation at ambient temperature for 10 min to ensure complete chromogenic reaction. The resulting optical density was measured at 540 nm using a microplate reader (Molecular Devices, Sunnyvale, CA, USA). Sample nitrite concentrations were calculated by interpolation from a linear standard curve constructed with serial dilutions of sodium nitrite standards [19].

### 2.9. The TBARS (Thiobarbituric Acid Reactive Substance) Assay

The extent of lipid peroxidation in small intestinal tissues was assessed by quantifying malondialdehyde (MDA) content, a well-established biomarker of thiobarbituric acid reactive substances (TBARS). Tissue samples were homogenized in ice-cold lysis buffer to obtain homogenates. The lysates were then incubated with thiobarbituric acid (TBA) reagent at 90 °C for 45 min to facilitate the formation of MDA-TBA adducts. The resulting chromogenic complexes were quantified by measuring absorbance at 532 nm using a spectrophotometer. MDA concentrations were calculated by interpolation from a standard curve prepared with known concentrations of 1,1,3,3-tetraethoxypropane (TEP), providing a reliable and sensitive index of oxidative lipid damage in the experimental tissues [18].

### 2.10. Glutathione (GSH) Assay

The content of reduced glutathione (GSH) was determined using Ellman’s colorimetric method with DTNB (5,5′-dithiobis-(2-nitrobenzoic acid)) as the chromogenic substrate. Reaction mixtures were prepared by combining 100 μL of sample supernatant, 200 μL of 0.3 M phosphate buffer (pH 8.4), 400 μL of double-distilled water, and 500 μL of Ellman’s reagent (DTNB solution). After gentle mixing and incubation, the absorbance was measured at 412 nm using a spectrophotometer. GSH concentrations were then normalized to protein content to facilitate accurate comparison and interpretation of antioxidant status across experimental groups [19].

### 2.11. Western Blot Analysis

Total protein was extracted from small intestinal tissues. Briefly, tissue samples (approximately 50–100 mg) were minced on ice and homogenized in 500 µL of ice-cold RIPA lysis buffer (25 mM Tris-HCl, pH 7.6, 150 mM NaCl, 1% NP-40, 1% sodium deoxycholate, 0.1% SDS) supplemented with a complete protease inhibitor cocktail (Roche, Basel, Switzerland) to minimize protein degradation. Homogenization was performed using a tissue homogenizer (IKA-Werke, Staufen, Germany) at 10,000 rpm for 30 s on ice. The homogenates were then incubated on ice for 30 min to allow complete lysis, followed by centrifugation at 12,000× *g* for 20 min at 4 °C. The resulting supernatant containing the total protein fraction was collected and transferred to a pre-chilled microcentrifuge tube. Protein concentrations were determined using the Bradford dye-binding assay. Briefly, aliquots of each sample were mixed with the Bradford reagent (Bio-Rad Laboratories, Hemel Hempstead, UK) according to the manufacturer’s protocol. After incubation at room temperature for 5 min, the absorbance was measured at 595 nm using a microplate reader (Model 680, Bio-Rad). A standard curve was generated using bovine serum albumin (BSA) as a standard, and protein concentrations in the samples were calculated by interpolation from the linear regression of the standard curve. All measurements were performed in duplicate. Western blotting was performed as previously described [19] with minor modifications. Briefly, equal amounts of protein (30 μg) were resolved by SDS-PAGE on 10% gels and transferred to PVDF membranes (Millipore, Burlington, MA, USA). Membranes were blocked with 5% skim milk in TBST and then probed with primary antibodies (1:1000) overnight at 4 °C, followed by HRP-conjugated secondary antibodies (1:5000) for 1 h at room temperature. Protein bands were visualized using enhanced chemiluminescence (ECL) (Thermo Fisher Scientific, Waltham, MA, USA) and captured with Kodak Molecular Imaging Software 5.0 (Eastman Kodak Company, Rochester, NY, USA). Densitometric analysis was performed using ImageJ 1.54 software (National Institutes of Health, Bethesda, MD, USA), with β-actin serving as the loading control.

### 2.12. Statistical Analysis

Results were analyzed statistically, with values presented as mean ± standard deviation (S.D.). Student’s *t*-test was employed for pairwise comparisons, and one-way ANOVA followed by Scheffé’s post hoc analysis was used to evaluate differences among multiple treatments. A threshold of *p* < 0.05 denoted statistical significance.

## 3. Results

### 3.1. The Modulatory Effects of EMC on Physical Manifestations Such as Weight Reduction, Diarrhea, and Changes in Intestinal Length Were Systematically Examined in Mice Subjected to 5-FU-Induced Intestinal Mucositis

**Figure 1:**
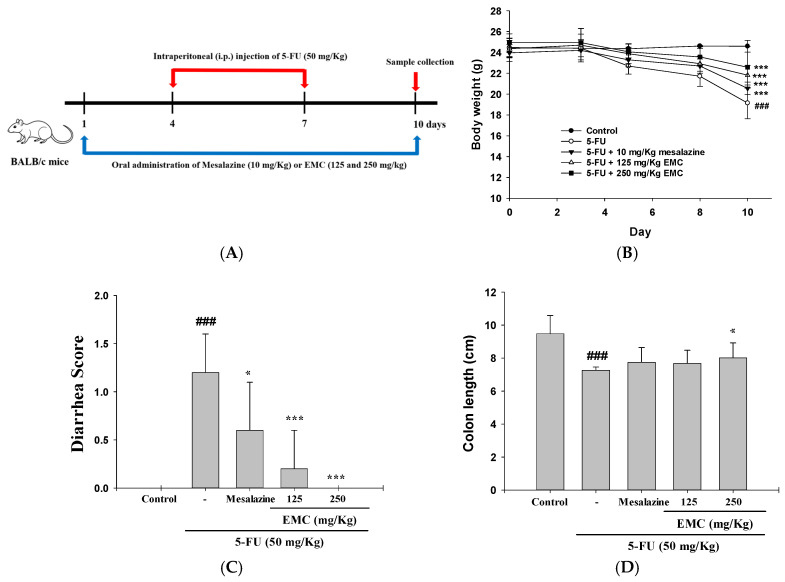
EMC administration alleviated intestinal mucositis induced by 5-FU in mice. The experimental setup (**A**) involved monitoring body weight (**B**), diarrhea severity (**C**), and intestinal length (**D**). EMC (125 or 250 mg/kg) was administered daily for 10 days, with 5-FU injection performed from days 4 to 7. Mice were sacrificed on day 10. Data are represented as mean ± S.D. (n = 5). ^###^ *p* < 0.001 vs. control; * *p* < 0.05 and *** *p* < 0.001 vs. 5-FU.

Body weight reduction and diarrhea were the primary clinical parameters used to assess the general health status of the experimental mice. Continuous monitoring of mucositis-associated symptoms, including piloerection, anorexia, dehydration, and reduced locomotor activity, was conducted throughout the experimental period. Mice in the control group exhibited stable body weight, smooth fur, normal food and water intake, and active behavior, with no observable signs of diarrhea or distress. In contrast, 5-FU–treated animals displayed marked weight loss, lethargy, and watery stools, consistent with the onset of intestinal mucositis. Figure 1B illustrates the time-dependent effects of 5-FU treatment on murine body weight. Initially, by day 5, 5-FU-treated mice displayed a modest weight reduction (22.7 ± 0.78 g) compared to controls (24.36 ± 0.14 g), which correlated with the onset of overt clinical symptoms, such as diarrhea, anorexia, hypoactivity, and poor grooming. The toxicity progressed rapidly; by day 8, symptoms intensified to include blood-tinged watery diarrhea and an arched back, foreshadowing further decline. This deterioration culminated on day 10, where 5-FU-treated mice reached a significantly lower body weight (19.14 ± 0.15 g) than their control counterparts (24.6 ± 0.56 g, *p* < 0.001), highlighting the severe cachectic impact of the chemotherapy regimen. However, pretreatment with EMC effectively ameliorated these effects in a dose-dependent pattern, as evidenced by substantial improvements at both 125 and 250 mg/kg. These findings indicate that EMC confers protective benefits during the onset and progression of 5-FU-induced intestinal mucosal injury (Figure 1B).

The severity of diarrhea was graded daily using the Bowen scoring system: 0 (normal), 1 (slightly soft), 2 (wet and shapeless), and 3 (watery). The ameliorative effect of EMC on 5-FU-induced enteromucosal inflammation is supported by quantitative evidence from day 10 recordings, which showed significantly lower diarrhea scores in the EMC treatment group compared to the 5-FU group (Figure 1C).

As an indicator of intestinal structural preservation, colon length was measured following treatment. The 5-FU group exhibited severe colon shortening (7.26 cm) relative to the control (9.48 cm), reflecting inflammation-induced tissue contraction and epithelial loss. However, pretreatment with EMC (250 mg/kg) significantly counteracted this pathological shortening, resulting in an average colon length of 8.02 cm. This morphological recovery implies that EMC mitigates chemotherapeutic intestinal toxicity by preserving epithelial architecture (Figure 1D). The present study was designed primarily to evaluate the protective effects of EMC against 5-FU-induced intestinal injury. Consequently, a control group receiving EMC alone (without 5-FU challenge) was not included, as the safety profile of EMC in healthy mice has been previously established. Prior evaluation of the in vivo effects of oral EMC, conducted using an established animal model, demonstrated that administration of EMC at 250 mg/kg for 10 consecutive days as a pretreatment did not induce significant alterations in mouse body weight [17]. These findings suggest that EMC does not exert intrinsic intestinal toxicity at the dosage employed in this study.

### 3.2. EMC Mitigates 5-FU-Induced Histopathological Alterations in Intestinal Tissues Through Protective Modulation

**Figure 2:**
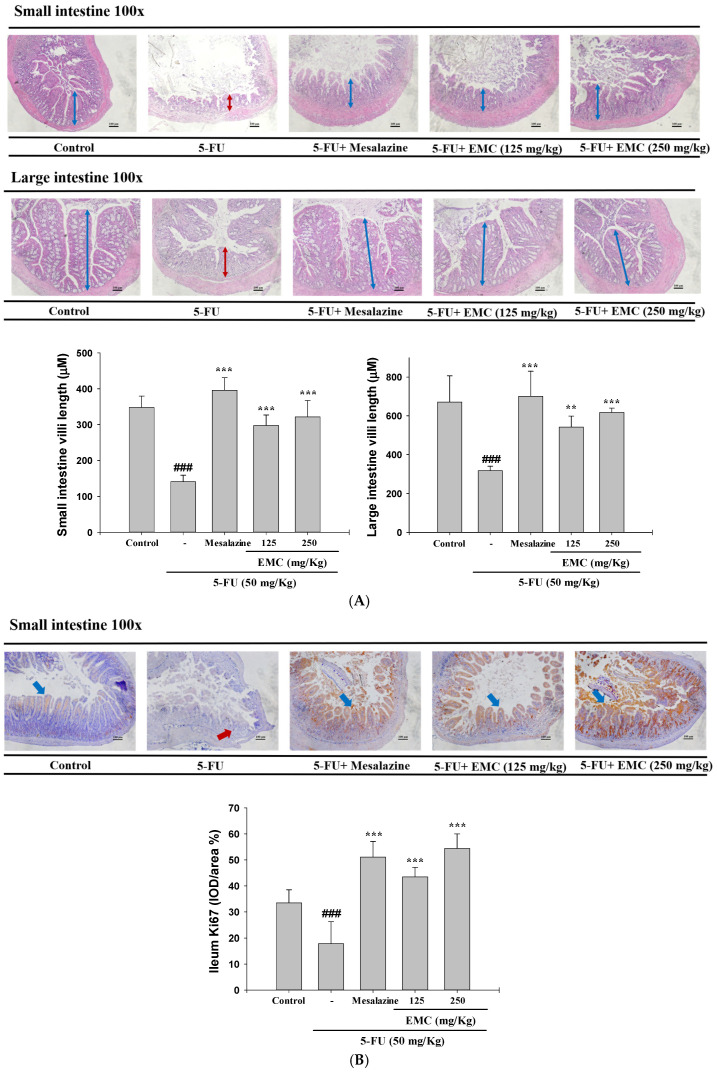
The influence of EMC on intestinal architecture and cell proliferation was evaluated through H&E (**A**) and Ki-67 (**B**) staining of tissues from 5-FU-exposed mice. Following daily EMC treatment (125 or 250 mg/kg) for 10 days and 5-FU administration during days 4–7, tissue samples were harvested on day 10 and analyzed microscopically for histopathological and immunohistochemical alterations. Data are represented as mean ± S.D. (n = 5). ^###^ *p* < 0.001 vs. control; ** *p* < 0.01, and *** *p* < 0.001 vs. 5-FU. Magnification, ×100; scale bar, 100 µm. Red and blue arrows indicate the sites where villus length (µm) was measured in the small and large intestine.

Intestinal mucosal integrity was assessed by hematoxylin and eosin (H&E) staining. The control group exhibited normal intestinal morphology, with elongated villi lined by healthy columnar epithelial and goblet cells, and crypts demonstrating regular mitotic activity. In sharp contrast, mice exposed to 5-FU displayed extensive mucosal deterioration, evidenced by total crypt depletion, villous collapse, vacuolar degeneration of epithelial cells, interstitial edema, and infiltration of inflammatory cells within the lamina propria. Such structural disintegration reflects severe epithelial loss and compromised barrier integrity resulting from 5-FU toxicity (Figure 2A). Pre-treatment with EMC mitigated morphological changes in the small and large intestines, reducing villus blunting, crypt cell apoptosis, and inflammatory cell infiltration. These findings suggest that EMC significantly alleviates 5-FU-induced damage in both intestinal regions.

### 3.3. Elevated Ki-67 Levels in the EMC-Treated Group Indicated Improved Cellular Proliferation in the Mucositis Model

Ki-67 staining demonstrated a clear distinction between groups. In the control group, intranuclear and cytoplasmic Ki-67 immunoreactivity was prominent in both the villus and crypt regions, indicating active epithelial proliferation. In stark contrast, 5-FU treatment, which targets rapidly dividing crypt epithelial cells, resulted in a significant reduction in Ki-67 expression (*p* < 0.01), reflecting impaired proliferative capacity and consequent mucosal barrier disruption. Remarkably, a significant increase in Ki-67 expression was demonstrated in the EMC-treated group, confirming the amelioration of this impairment (Figure 2B).

### 3.4. Administration of EMC Markedly Reduced the Upregulation of Pro-Inflammatory Cytokines Induced by 5-FU Exposure

**Figure 3:**
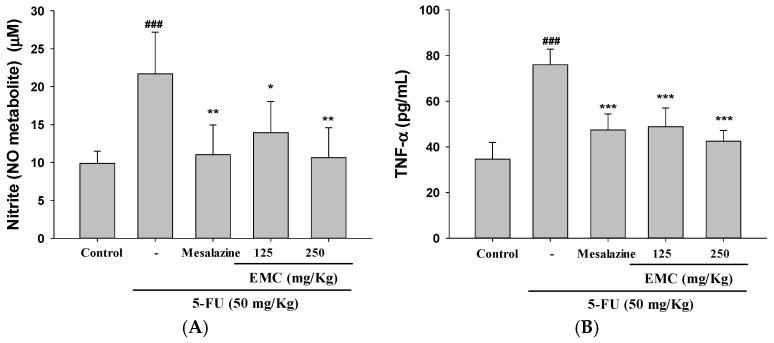

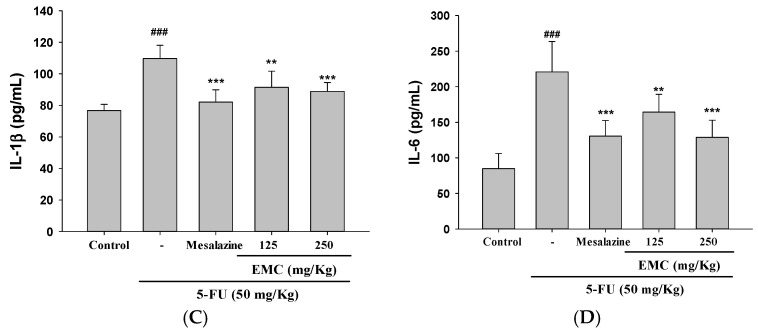
EMC administration resulted in a pronounced mitigation of inflammation in mice with 5-FU-induced intestinal mucositis, as indicated by significantly lower serum levels of nitrite (NO metabolite) (**A**), TNF-α (**B**), IL-1β (**C**), and IL-6 (**D**). Daily dosing of EMC (250 or 500 mg/kg) was performed for 10 consecutive days, whereas 5-FU was administered from days 4 through 7. Mice were euthanized on day 10 for biochemical evaluation. Data are represented as mean ± S.D. (n = 5). ^###^ *p* < 0.001 vs. control; * *p* < 0.05, ** *p* < 0.01, and *** *p* < 0.001 vs. 5-FU.

To elucidate the molecular mechanisms underlying the protective effects of EMC against 5-FU-induced intestinal mucositis, we examined the expression profiles of key inflammatory mediators in the small intestine of treated mice. Given that mucositis pathogenesis is largely driven by excessive inflammatory signaling, modulation of pro-inflammatory cytokine production represents a critical therapeutic target. As shown in Figure 3A–D, 5-FU administration resulted in a pronounced elevation in serum levels of nitrite [nitric oxide (NO) metabolite, tumor necrosis factor-α (TNF-α), interleukin (IL)-1β, and IL-6 levels, indicating the activation of downstream inflammatory cascades. In contrast, pretreatment with EMC at doses of 250 and 500 mg/kg resulted in significantly lower levels of these cytokines compared to the 5-FU group. This anti-inflammatory effect was comparable to that of mesalazine (10 mg/kg), a standard reference drug used for intestinal inflammation. These findings point to a mechanism whereby EMC inhibits inflammatory mediators through interference with redox-sensitive signaling and transcription factor activity. Subsequent experiments will validate whether EMC counteracts 5-FU-induced oxidative stress by enhancing antioxidant defenses and modulating the TLR4/IKK/NF-κB and HO-1/Nrf2 pathways.

### 3.5. Oxidative Stress Induced by 5-FU Was Significantly Reduced by EMC Treatment

**Figure 4:**
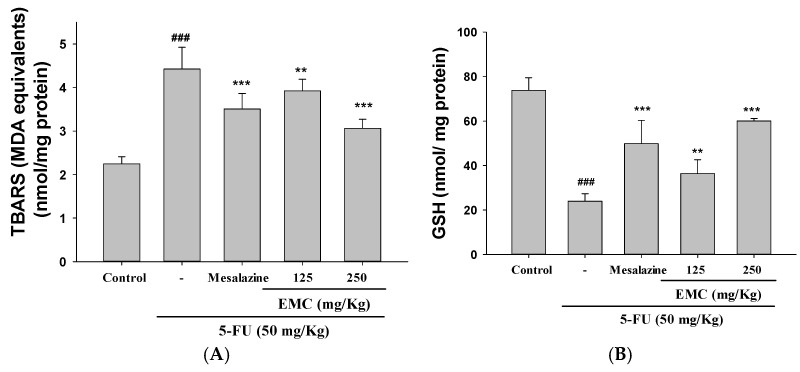
Measurements of TBARS (MDA equivalents) (**A**) and GSH (**B**) indicated that EMC treatment significantly reduced oxidative stress in the intestinal mucosa damaged by 5-FU. Data are represented as mean ± S.D. (n = 5). ^###^ *p* < 0.001 vs. control; ** *p* < 0.01, and *** *p* < 0.001 vs. 5-FU.

ROS overproduction is a key pathological factor driving oxidative stress and apoptosis. The antioxidative potential of EMC in 5-FU-induced intestinal injury was evaluated by measuring thiobarbituric acid reactive substances (TBARS) (expressed as malondialdehyde [MDA] equivalents) and glutathione (GSH) levels in the mouse small intestine. 5-FU treatment resulted in a marked increase in MDA levels relative to the control group. However, this elevation was significantly reduced by EMC pretreatment at both 125 and 250 mg/kg, indicating that EMC attenuates 5-FU-induced oxidative stress (Figure 4A).

To further delineate the antioxidative mechanism of EMC, intracellular GSH levels were quantified in intestinal tissues. Exposure to 5-FU resulted in a pronounced depletion of GSH, signifying compromised redox homeostasis. EMC administration (125 and 250 mg/kg) markedly increased GSH levels, suggesting that its ROS-scavenging activity enhances endogenous antioxidant enzyme function. These results underscore EMC’s mechanistic role in preserving redox equilibrium and protecting intestinal mucosa from chemotherapy-induced oxidative stress (Figure 4B).

### 3.6. EMC Treatment Effectively Reduced Inflammation by Downregulating the NF-κB and MAPK Signaling Cascades in Mice with 5-FU-Induced Intestinal Mucositis

**Figure 5:**
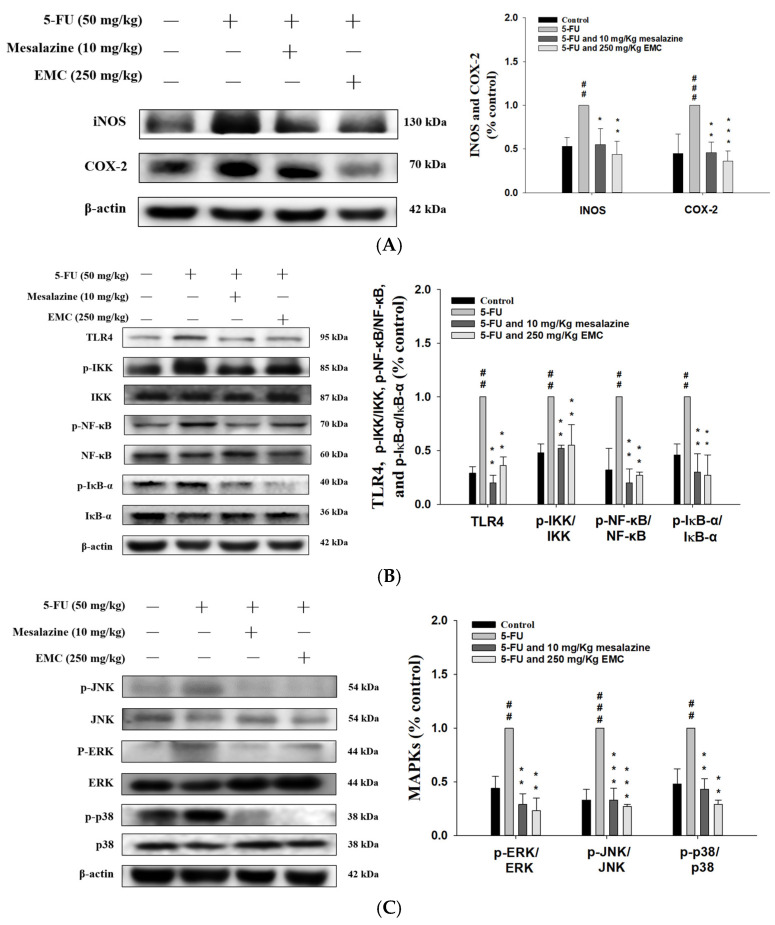
EMC administration reduced the activation of major inflammatory pathways in the 5-FU-induced mucositis model, as reflected by the downregulation of iNOS and COX-2 (**A**), the TLR4/IKK/NF-κB axis (**B**), and phosphorylated MAPK proteins (**C**). Homogenized small intestinal lysates were analyzed via Western blot to assess the protein expression. The bar chart illustrates that protein bands were quantified by densitometry, normalized against β-actin, and subsequently expressed as the relative ratio to the 5-FU-induced group. The value of the 5-FU-induced group was defined as 1.0 for comparison. Densitometric quantification was performed to determine the ratio of phosphorylated (activated) to total (non-activated) protein levels for key components of the signaling cascade, including IKK, NF-κB, IκB-α, and MAPKs (ERK1/2, JNK, and p38). Data are presented as mean ± S.D. of at least three independent experiments. Statistical significance is denoted as follows: ^###^ *p* < 0.001 and ^##^ *p* < 0.01 compared to the control group; *** *p* < 0.001, ** *p* < 0.01, and * *p* < 0.05 compared to the 5-FU group.

Inflammation represents a complex biological response involving the activation of diverse molecular pathways and transcriptional regulators that orchestrate cytokine release, immune cell recruitment, and tissue remodeling [18]. In the present study, EMC (250 mg/kg) treatment exhibited pronounced anti-inflammatory effects, as evidenced by the marked downregulation of COX-2 expression in the intestinal tissues of 5-FU-treated mice (Figure 5A). Similarly, the expression of iNOS, a key pro-inflammatory enzyme implicated in NO overproduction and mucosal injury, was significantly reduced following EMC administration. These inhibitory effects were comparable to those observed in the mesalazine-treated positive control group, an outcome that validates the experimental model and confirms the anti-inflammatory efficacy of EMC.

Toll-like receptors (TLRs) play a pivotal role in the innate immune system by detecting pathogen-associated molecular patterns (PAMPs) and initiating downstream immune signaling [19]. In this study, 5-FU administration markedly enhanced TLR4 activation, as evidenced by Western blot analysis (Figure 5B). Pretreatment with EMC significantly mitigated this upregulation, indicating its capacity to modulate TLR4-dependent inflammatory cascades. The attenuation of TLR4 signaling by EMC suggests that its anti-inflammatory efficacy may be mediated through the reduction in this receptor expression, thereby dampening mucosal inflammation. Collectively, these findings highlight the regulatory potential of EMC in targeting TLR4-driven inflammatory responses during 5-FU-induced intestinal mucositis.

The Nuclear Factor-κB (NF-κB) signaling pathway plays a central role in orchestrating inflammatory cascades and is widely implicated in the pathogenesis of inflammatory diseases [18]. Our findings showed that 5-FU markedly upregulated the phosphorylated forms of NF-κB, inhibitory-κB kinase (IKK), and NF-κB inhibitor α (IκB-α) in small intestinal tissues. Notably, EMC pretreatment effectively counteracted these effects, reducing the phosphorylation of these key mediators in 5-FU-induced intestinal injury. These data demonstrate that EMC reduces inflammation primarily through modulation of the TLR4/IKK/NF-κB signaling axis (Figure 5B).

The mitogen-activated protein kinase (MAPK) pathway is an essential regulator of inflammatory signaling and plays a central role in the development of many inflammatory disorders [19]. In this experiment, 5-FU treatment induced robust phosphorylation of JNK, ERK, and p38, demonstrating MAPK pathway activation in intestinal mucosal tissues. Notably, pretreatment with EMC and mesalazine significantly decreased the phosphorylated (p-ERK1/2, p-JNK, and p-p38)/total ratio of MAPKs (ERK1/2, JNK, and p38). This finding suggests that the inhibition is mediated at the post-translational level. Collectively, these findings highlight that EMC mitigates 5-FU-induced inflammation through selective inhibition of MAPK phosphorylation, thereby preserving intestinal tissue integrity (Figure 5C).

### 3.7. Treatment with EMC Strengthened the Intestinal Antioxidant Defense System and Stimulated the HO-1/Nrf2 Pathway, Thereby Counteracting Oxidative Stress Induced by 5-FU

**Figure 6:**
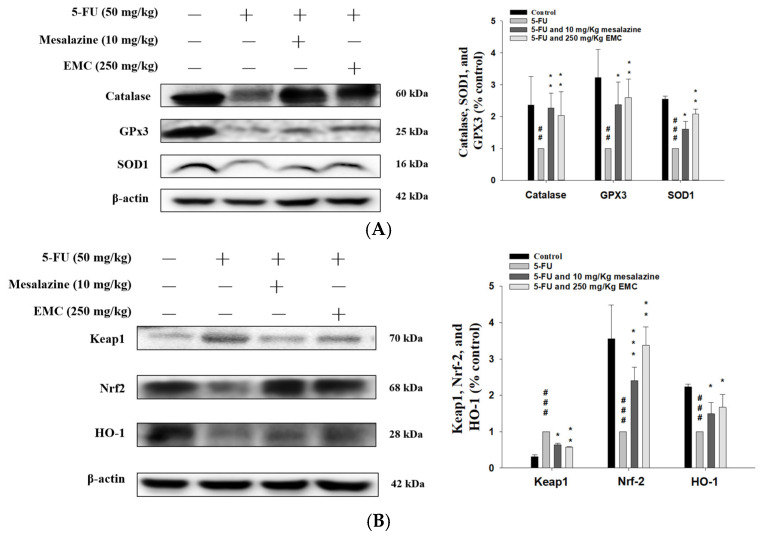
EMC effectively counteracted 5-FU-induced oxidative dysregulation by modulating antioxidative enzyme expression (catalase, SOD1, GPx3) (**A**) and components of the Keap1/Nrf2/HO-1 axis (**B**). Homogenized small intestinal lysates were analyzed via Western blot to assess the protein expression. The bar chart illustrates that protein bands were quantified by densitometry, normalized against β-actin, and subsequently expressed as the relative ratio to the 5-FU-induced group. The value of the 5-FU-induced group was defined as 1.0 for comparison. Data are presented as mean ± S.D. of at least three independent experiments. Statistical significance is denoted as follows: ^###^ *p* < 0.001 and ^##^ *p* < 0.01 compared to the control group; *** *p* < 0.001, ** *p* < 0.01, and * *p* < 0.05 compared to the 5-FU group.

Oxidative stress represents a critical pathological event that compromises intestinal function and initiates inflammatory cascades, leading to progressive mucosal injury [19]. In this study, exposure to 5-FU significantly downregulated catalase, SOD1, and GPx3 in the small intestine (Figure 6A), reflecting an impaired antioxidant defense network. Remarkably, EMC pretreatment improved these antioxidant enzyme levels, implying its regulatory effect on oxidative signaling and its potential to prevent oxidative damage-mediated inflammation.

Oxidative stress plays a pivotal role in intestinal mucosal injury by promoting excessive ROS generation, which in turn damages lipids, nucleic acids, and essential macromolecules, ultimately leading to apoptotic and necrotic cell death [19]. In the current study, 5-FU administration markedly elevated Keap1 expression while concurrently reducing HO-1 and Nrf2 protein levels in intestinal tissues compared to the control group (Figure 6B), indicating impaired antioxidant defense. Notably, compared to mice treated with 5-FU alone, pretreatment with EMC partly countered the 5-FU-induced alterations by increasing Nrf2 and HO-1 expression and reducing Keap1 upregulation (Figure 6B). These molecular changes suggest that EMC activates the Nrf2/HO-1 signaling pathway, thereby enhancing endogenous antioxidant capacity and mitigating oxidative damage induced by 5-FU exposure.

### 3.8. EMC Attenuates the Activation of Apoptosis-Related Signaling Pathways Triggered by 5-FU Treatment

**Figure 7:**
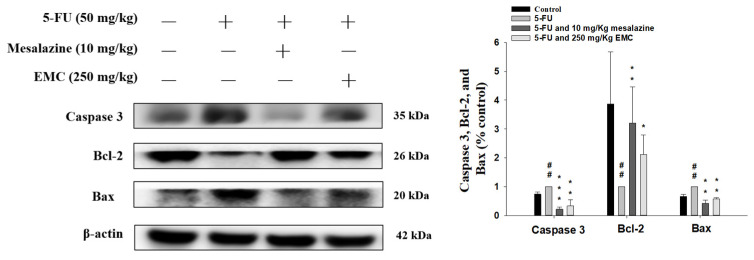
EMC treatment notably influenced the apoptotic profile of small intestinal tissues exposed to 5-FU, as evidenced by altered expression of Bax, Bcl-2, and caspase-3. Homogenized small intestinal lysates were analyzed via Western blot to assess the protein expression. The bar chart illustrates that protein bands were quantified by densitometry, normalized against β-actin, and subsequently expressed as the relative ratio to the 5-FU-induced group. The value of the 5-FU-induced group was defined as 1.0 for comparison. Data are presented as mean ± S.D. of at least three independent experiments. Data are presented as mean ± S.D. of at least three independent experiments. Statistical significance is denoted as follows: ^##^ *p* < 0.01 compared to the control group; *** *p* < 0.001, ** *p* < 0.01, and * *p* < 0.05 compared to the 5-FU group.

5-FU induces apoptosis signaling pathways by causing irreversible DNA damage and RNA dysfunction, ultimately activating both intrinsic and extrinsic apoptosis pathways [18]. Immunoblotting assays confirmed that exposure to 5-FU triggered apoptotic signaling, as shown by elevated Bax and caspase-3 expression and diminished Bcl-2 levels. Remarkably, EMC pretreatment abrogated these apoptotic effects, reinstating Bcl-2 expression and reducing Bax and caspase-3 activation. These findings highlight EMC’s efficacy in mitigating chemotherapy-induced intestinal epithelial apoptosis through modulation of intrinsic apoptotic pathways (Figure 7).

### 3.9. The 5-FU-Induced Alterations in the GSK3β/Cyclin D1 Signaling Cascade Were Markedly Attenuated Following EMC Administration

**Figure 8:**
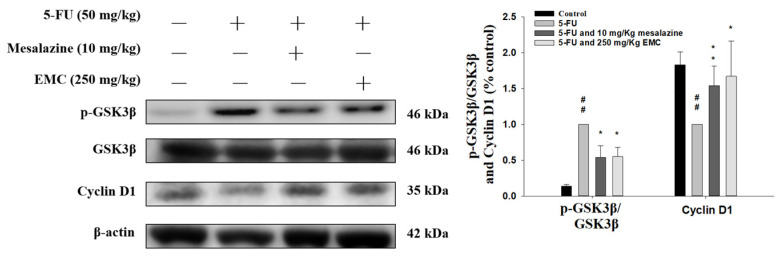
EMC mitigated 5-FU-induced alterations in the GSK3β/cyclin D1 signaling pathway. Homogenized small intestinal lysates were analyzed via Western blot to assess the protein expression. The bar chart illustrates that protein bands were quantified by densitometry, normalized against β-actin, and subsequently expressed as the relative ratio to the 5-FU-induced group. The value of the 5-FU-induced group was defined as 1.0 for comparison. Densitometric quantification was performed to determine the ratio of phosphorylated (activated) to total (non-activated) GSK3β protein levels for key components of the signaling cascade. Data are presented as mean ± S.D. of at least three independent experiments. Data are presented as mean ± S.D. of at least three independent experiments. Statistical significance is denoted as follows: ^##^ *p* < 0.01 compared to the control group; ** *p* < 0.01, and * *p* < 0.05 compared to the 5-FU group.

The GSK3β/cyclin D1 axis serves as a crucial regulatory pathway governing cell cycle progression and epithelial proliferation. GSK-3β phosphorylates cyclin D1, targeting it for proteasomal degradation, thereby restricting excessive cellular growth [19]. As illustrated in Figure 8, compared with the control group, mice treated with 5-FU exhibited a pronounced elevation in p-GSK3β levels. In contrast, cyclin D1 protein expression was significantly decreased, indicating aberrant regulation of cell cycle-related signaling under chemotherapeutic stress. Notably, EMC pretreatment attenuated the 5-FU-induced dysregulation of the GSK3β/cyclin D1 axis, as evidenced by decreased p-GSK3β and increased cyclin D1 expression. This modulation suggests that EMC stabilizes epithelial homeostasis by attenuating dysregulated proliferative signaling. Collectively, these findings imply that EMC mitigates 5-FU-induced intestinal mucositis, at least in part, through the normalization of the GSK3β/cyclin D1 pathway, thereby contributing to epithelial recovery and maintenance of mucosal integrity.

### 3.10. EMC Reduces the Epithelial–Mesenchymal Transition (EMT) Signaling Cascade Triggered by 5-FU Exposure

**Figure 9:**
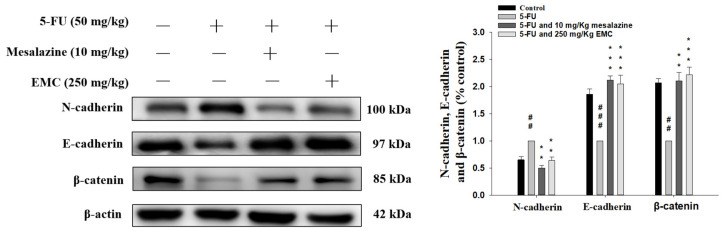
EMC treatment modulated the expression of EMT-associated proteins in mice with 5-FU-induced intestinal mucositis. Homogenized small intestinal lysates were analyzed via Western blot to assess the protein expression. The bar chart illustrates that protein bands were quantified by densitometry, normalized against β-actin, and subsequently expressed as the relative ratio to the 5-FU-induced group. The value of the 5-FU-induced group was defined as 1.0 for comparison. Data are presented as mean ± S.D. of at least three independent experiments. Statistical significance is denoted as follows: ^###^ *p* < 0.001 and ^##^ *p* < 0.01 compared to the control group; *** *p* < 0.001, and ** *p* < 0.01 compared to the 5-FU group.

EMT signaling pathways are increasingly recognized as critical mediators in the pathogenesis of 5-FU-induced intestinal mucositis, a condition characterized by extensive epithelial barrier disruption and impaired tissue regeneration. EMT involves the loss of epithelial integrity—manifested by diminished expression of cell adhesion molecules such as E-cadherin and β-catenin—concurrent with the acquisition of mesenchymal characteristics, including the upregulation of N-cadherin and enhanced migratory capacity [19]. As illustrated in Figure 9, 5-FU administration markedly reduced β-catenin and E-cadherin protein levels while promoting N-cadherin expression, indicative of EMT activation and epithelial destabilization. Notably, pretreatment with EMC effectively counteracted these alterations, increasing β-catenin and E-cadherin expression while reducing N-cadherin induction when compared to 5-FU. These findings highlight EMC’s potential to mitigate mucosal injury by modulating EMT-related pathways, thereby preserving epithelial architecture and facilitating mucosal recovery. Collectively, the data suggest that inhibition of EMT progression may represent a key mechanism underlying EMC’s protective action against chemotherapy-induced intestinal damage.

### 3.11. The Administration of EMC Attenuates Alterations in Tight Junction Protein Expression Caused by 5-FU

**Figure 10:**
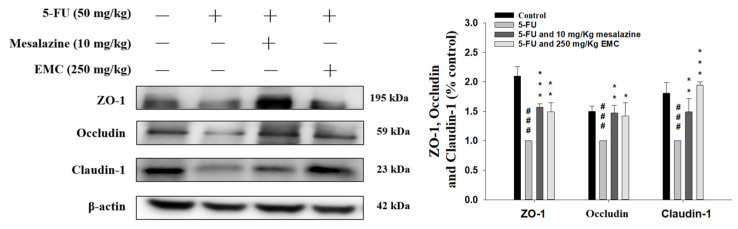
EMC treatment modulated the expression of tight junction proteins in mice exposed to 5-FU. Homogenized small intestinal lysates were analyzed via Western blot to assess the protein expression. The bar chart illustrates that protein bands were quantified by densitometry, normalized against β-actin, and subsequently expressed as the relative ratio to the 5-FU-induced group. The value of the 5-FU-induced group was defined as 1.0 for comparison. Data are presented as mean ± S.D. of at least three independent experiments. Statistical significance is denoted as follows: ^###^ *p* < 0.001 compared to the control group; *** *p* < 0.001, ** *p* < 0.01 and * *p* < 0.05 compared to the 5-FU group.

5-FU-induced mucositis severely compromises intestinal epithelial integrity by disrupting tight junction architecture, a key determinant of mucosal barrier function. Tight junction proteins, including ZO-1, occludin, and claudin-1, play pivotal roles in maintaining epithelial cohesion and regulating paracellular permeability [19]. As shown in Figure 10, 5-FU administration markedly decreased the expression of these structural proteins, leading to impaired barrier function and enhanced epithelial leakage. Remarkably, pretreatment with EMC markedly increased the expression of the tight junction proteins ZO-1, occludin, and claudin-1, with a concomitant reestablishment of epithelial continuity. This restoration suggests that EMC not only protects against chemotherapeutic damage but also promotes mucosal repair by stabilizing tight junction complexes. Collectively, these findings highlight EMC’s therapeutic potential in preserving epithelial barrier integrity and mitigating the pathological consequences of 5-FU-induced mucositis through modulation of tight junction-associated signaling pathways.

## 4. Discussion

Despite its established efficacy against solid tumors, the clinical utility of 5-FU is severely compromised by its off-target toxicity. Its mechanism of action—inhibition of thymidylate synthase—while effective against neoplasms, indiscriminately affects all rapidly proliferating cell populations [10]. This lack of selectivity predictably leads to dose-limiting toxicities in the gastrointestinal tract, bone marrow, and skin. Although the pathophysiological consequences (e.g., mucositis, myelosuppression) are well-documented, current clinical management remains predominantly supportive, addressing symptoms rather than the underlying mechanisms of cellular injury. This persistent therapeutic gap underscores the critical need for interventions that can selectively protect normal tissues [4]. The development of such interventions relies heavily on preclinical models that recapitulate human pathology. The dose-dependent nature of 5-FU injury in murine models offers a valuable tool for this purpose. While high-dose regimens effectively model acute toxicity, they often obscure the subtle, long-term protective effects of candidate agents. Conversely, the low-dose (≤50 mg/kg) regimen, as employed in this study, provides a more nuanced platform. It induces measurable clinical symptoms—such as weight loss and diarrhea—without compromising survival, allowing for a longitudinal assessment of therapeutic efficacy [20]. Our finding that EMC significantly attenuated diarrhea severity in this model is particularly compelling. It suggests that EMC targets a fundamental aspect of the pathological cascade, positioning it as a promising candidate for further mechanistic and translational investigation.

Although the dosing of 5-FU is meticulously adjusted according to individual patient parameters to optimize its therapeutic index, the occurrence of gastrointestinal mucositis remains a pervasive clinical challenge. This toxicity, manifesting as diarrhea, malabsorption, and pain, is not merely an incidental discomfort but a dose-limiting complication with significant ramifications [21]. Consequently, the resulting dose modifications or treatment interruptions directly undermine the curative intent of the therapy. Furthermore, the economic burden associated with managing these complications underscores the inadequacy of current supportive measures. From a pathophysiological standpoint, the disruption of intestinal epithelial homeostasis is central to this syndrome. The selective vulnerability of rapidly dividing crypt cells to 5-FU initiates a cascade of barrier failure, inflammatory activation, and oxidative damage that perpetuates mucosal injury [22]. This mechanistic understanding reveals a critical therapeutic gap: while the consequences of mucositis are well-characterized, interventions that directly target these fundamental pathological processes remain scarce. Thus, the development of agents capable of preserving intestinal integrity without compromising antitumor activity represents a pressing and unmet medical need.

Chemotherapy-induced diarrhea represents a prevalent and debilitating adverse effect, affecting approximately 50–80% of patients and often necessitating treatment interruption. In the present study, administration of 5-FU induced severe intestinal mucositis, characterized by villous stem cell apoptosis, reduced epithelial proliferation, and subsequent villus shortening [4]. Body weight began to decline on the fifth day of 5-FU treatment, reflecting systemic toxicity and nutrient malabsorption associated with mucosal injury. In contrast, mice receiving combined 5-FU and EMC treatment exhibited a gradual recovery in body weight from days 5 to 10, suggesting that EMC mitigates 5-FU-induced gastrointestinal toxicity. Notably, while profuse diarrhea was observed in the 5-FU-only group, it was completely absent in the 5-FU + EMC (500 mg/kg) group, underscoring the compound’s potent antidiarrheal and intestinal-protective effects. Histopathological assessment further substantiated these findings. H&E-stained intestinal sections from 5-FU-treated mice revealed pronounced villous atrophy, crypt destruction, and mucosal inflammation, all of which were markedly ameliorated by EMC administration. Furthermore, Immunohistochemical staining for Ki-67 was performed on intestinal tissue sections to quantify the impact of 5-FU on epithelial cell proliferation in the mucositis model. Ki-67 IHC analysis is a pivotal validation endpoint, as reduced Ki-67-positive proliferative crypt cells are directly linked to the model’s defining histopathological features, including crypt dysplasia, villous atrophy, and compromised barrier integrity [18]. This technique is therefore indispensable for model validation and for evaluating therapeutics intended to mitigate chemotherapy-induced epithelial injury and promote crypt regeneration. In tissues from 5-FU-treated mice, the number of Ki-67-positive nuclei within crypts was significantly and quantitatively decreased, with proliferative zones markedly reduced in size or fragmented. The absence of Ki-67 staining provides direct histological evidence of the antimetabolite mechanism of 5-FU, which inhibits DNA synthesis and arrests the cell cycle in rapidly dividing crypt epithelial cells [19]. Importantly, Ki-67 expression remained intact in non-proliferative, differentiated epithelial cells along the villi, demonstrating the spatial specificity of the drug’s cytotoxic effect. Immunohistochemical analysis in the EMC-treated group revealed a significant restoration of Ki-67-positive proliferative activity, indicating recovery of epithelial renewal processes impaired by 5-FU. Collectively, these results demonstrate that EMC effectively alleviates the clinical and histological manifestations of 5-FU-induced intestinal mucositis. Its capacity to prevent diarrhea, restore mucosal architecture, and enhance epithelial regeneration suggests a dual mechanism involving cytoprotection and promotion of intestinal repair [19]. These findings position EMC as a promising candidate for adjunctive therapy to counteract chemotherapy-induced intestinal toxicity.

Maintenance of mucosal homeostasis relies on a delicate balance of cytokines produced by the intestinal immune system, which regulate immune cell activity, epithelial barrier function, and microbial equilibrium. Disruption of this balance, as observed following 5-FU administration, leads to excessive pro-inflammatory cytokine production and the development of mucositis [21]. In this study, EMC treatment effectively reduced 5-FU-induced upregulation of TNF-α, IL-1β, and IL-6, thereby attenuating cytokine-driven mucosal injury and contributing to the restoration of immune balance. This immunomodulatory effect, coupled with the known involvement of oxidative pathways in chemotherapy-induced mucositis, suggests a dual protective mechanism involving the attenuation of pro-inflammatory signaling and the preservation of epithelial integrity [22]. A central mechanism driving 5-FU-induced intestinal injury is the establishment of a vicious cycle between oxidative stress and inflammation. 5-FU generates excessive ROS, causing oxidative damage to DNA, lipids, and proteins, which triggers epithelial apoptosis and barrier disruption. This initial injury activates innate immune responses, leading to the release of pro-inflammatory cytokines (TNF-α, IL-1β, IL-6) that amplify tissue inflammation, increase vascular permeability, and promote leukocyte infiltration. Concurrently, cytokine-mediated inflammation further accelerates ROS production, creating a self-perpetuating loop that exacerbates mucosal damage, tight junction dysregulation, and clinical symptoms such as diarrhea [23]. TNF-α plays a particularly critical role in amplifying this cascade. Our findings demonstrate that EMC effectively interrupts this detrimental cycle by reducing pro-inflammatory cytokine levels and attenuating the activation of associated signaling pathways. Given the established role of oxidative stress in mucositis, these effects may also involve modulation of redox balance, a hypothesis warranting further investigation. By breaking the oxidative–inflammatory crosstalk, EMC promotes mucosal healing and mitigates 5-FU-induced intestinal injury [24].

As a pivotal component of the innate immune surveillance system, TLR4 detects both pathogen-associated molecular patterns (PAMPs) and damage-associated molecular patterns (DAMPs), subsequently triggering intracellular signaling cascades that culminate in the production of inflammatory mediators [25]. Upon ligand engagement, TLR4 recruits adaptor proteins such as MyD88, leading to the phosphorylation and nuclear translocation of NF-κB p65, thereby inducing the transcription of pro-inflammatory cytokines and enzymes, including TNF-α, IL-6, IL-1β, COX-2, and iNOS [26]. In the context of 5-FU-induced intestinal mucositis, aberrant activation of the TLR4/NF-κB signaling axis results in excessive cytokine release within the intestinal mucosa, disrupting epithelial integrity and exacerbating mucosal injury. Elevated levels of TNF-α, IL-6, and IL-1β in the ileum correlate with enhanced infiltration of inflammatory cells and epithelial apoptosis, further perpetuating tissue damage [27]. Importantly, pharmacological intervention targeting TLR4/NF-κB signaling—such as treatment with EMC—can reduce p65 phosphorylation and cytokine production, thereby restoring mucosal homeostasis. These findings highlight the central role of TLR4-mediated signaling in the initiation and amplification of mucosal inflammation, and suggest that inhibition of this pathway represents a viable strategy for mitigating chemotherapy-induced mucosal toxicity. These results underscore the identification of potential therapeutic targets for mitigating mucositis severity in clinical contexts. Enhanced NF-κB activation represents a defining feature of inflammation, cytokine overproduction, and mucosal injury, as evidenced by histopathological analyses of the small intestine. Mice treated with 5-FU exhibited markedly elevated TNF-α, IL-6, and IL-1β levels relative to controls [18]. Pretreatment with EMC significantly attenuated cytokine release and NF-κB activation while preserving mucosal architecture. Collectively, these findings suggest that EMC exerts its protective actions by reducing TLR4 activation and inhibiting NF-κB p65 phosphorylation.

MAPKs play a pivotal role in the pathogenesis of 5-FU-induced intestinal mucositis. This family of serine/threonine kinases—including extracellular signal-regulated kinase (ERK), c-Jun N-terminal kinase (JNK), and p38 MAPK—functions as a critical signaling hub that translates extracellular stress cues into cellular responses [28]. These kinases are rapidly activated in response to oxidative stress, proinflammatory cytokines, and chemotherapeutic insults such as 5-FU. Once activated, MAPKs orchestrate a broad spectrum of cellular processes, including inflammation, apoptosis, and tissue regeneration. In experimental models of 5-FU-induced mucositis, enhanced phosphorylation of MAPKs has been consistently associated with mucosal inflammation, crypt destruction, and epithelial barrier dysfunction [29]. Mechanistically, MAPK activation amplifies inflammatory cascades by promoting the expression of key mediators, such as NF-κB, TNF-α, IL-1β, and COX-2. Notably, the p38 and JNK MAPK subtypes have been shown to facilitate NF-κB activation through phosphorylation of upstream regulatory proteins, including IκB kinase (IKK), thereby sustaining a feed-forward inflammatory loop [30]. Given their central role in regulating both inflammatory and apoptotic pathways, MAPKs represent promising therapeutic targets for attenuating chemotherapy-induced mucosal injury. Pharmacological inhibition or phytochemical modulation of MAPK signaling has been demonstrated to reduce cytokine production, oxidative stress, and epithelial apoptosis, highlighting the translational potential of targeting these kinases to preserve intestinal homeostasis during 5-FU chemotherapy [18]. MAPK signaling directly governs the transcriptional activation and release of TNF-α, a crucial proinflammatory cytokine implicated in the initiation and persistence of mucositis. Excessive ROS generated during 5-FU treatment further stimulate MAPK activation, which subsequently amplifies NF-κB and COX-2 pathways, perpetuating an oxidative–inflammatory feedback loop [19]. The current results suggest that EMC exerts a protective effect against 5-FU-induced mucosal injury primarily by attenuating the phosphorylation of MAPKs and inhibiting NF-κB activation. By downregulating pro-inflammatory cytokine expression, EMC demonstrates strong anti-inflammatory potential. Thus, modulation of the NF-κB/MAPK axis may underlie the therapeutic efficacy of EMC in managing chemotherapy-associated mucositis.

5-FU-induced mucositis is strongly associated with oxidative stress, which acts as a principal mediator of cellular injury and inflammation during chemotherapy. As part of its cytotoxic mechanism, 5-FU promotes excessive ROS production, effectively targeting rapidly proliferating tumor cells but inadvertently damaging normal tissues, especially the intestinal epithelium, characterized by high turnover rates [31]. The resulting accumulation of ROS overwhelms endogenous antioxidant defenses, including GSH, catalase, and SOD, thereby disrupting redox homeostasis. This oxidative imbalance triggers a cascade of deleterious events—lipid peroxidation, DNA fragmentation, and protein carbonylation—culminating in epithelial apoptosis, tight junction disintegration, and compromised mucosal integrity [19]. The Nrf2/HO-1 signaling axis represents a crucial adaptive mechanism against oxidative stress, orchestrating the transcriptional activation of antioxidant and cytoprotective genes. However, oxidative stimuli induce Nrf2 dissociation, nuclear translocation, and subsequent activation of downstream effectors such as HO-1, NAD(P)H-Quinone Oxidoreductase 1 (NQO1), and glutamate–cysteine ligase catalytic subunit (GCLC) [32]. In the context of 5-FU-induced mucositis, dysregulation of this pathway exacerbates oxidative injury and inflammatory responses. Restoration or pharmacological activation of the Nrf2 pathway has thus emerged as a promising therapeutic strategy to mitigate mucosal damage, enhance epithelial resilience, and preserve intestinal barrier function during chemotherapy [33]. Conversely, excessive ROS accumulation triggers the activation of the MAPK signaling cascade, which in turn promotes the expression of inflammatory mediators and pro-apoptotic factors, thereby exacerbating epithelial injury in intestinal tissues. Moreover, oxidative stress perpetuates inflammation through the enhanced release of pro-inflammatory cytokines, including TNF-α, IL-6, and IL-1β, establishing a vicious cycle that sustains mucosal damage and amplifies oxidative imbalance. Concurrently, oxidative stress-induced activation of the TLR4/NF-κB/MAPK pathway further augments inflammatory signaling, resulting in aggravated mucosal disruption and compromised barrier integrity [34]. Emerging evidence suggests that oxidative stress not only induces histopathological deterioration via lipid peroxidation but also reduces endogenous antioxidant defense systems, thus aggravating mucosal vulnerability to chemotherapeutic injury [7]. Consistent with these findings, our experimental data demonstrate that EMC exerts protective effects by modulating the Nrf2-HO-1 signaling axis, thereby attenuating oxidative stress, mitigating inflammatory responses, and preserving intestinal structural integrity under 5-FU-induced challenge.

Apoptosis represents a central pathological mechanism driving the development of 5-FU-induced intestinal mucositis. Although 5-FU exerts potent cytotoxic effects against rapidly proliferating malignant cells, its non-selective inhibition of DNA and RNA synthesis in normal intestinal epithelial cells leads to severe mucosal injury [35]. The resultant crypt cell depletion, villus atrophy, and diminished regenerative potential collectively compromise mucosal integrity. Consequently, epithelial barrier dysfunction facilitates increased intestinal permeability, bacterial translocation, and nutrient malabsorption, which clinically manifest as diarrhea, weight loss, and systemic inflammation [36]. In the present study, EMC pretreatment markedly attenuated these deleterious effects, as evidenced by a pronounced downregulation of the pro-apoptotic proteins Bax and cleaved caspase-3, alongside the restoration of the anti-apoptotic factor Bcl-2 [37]. This modulation of the intrinsic apoptotic pathway suggests that EMC confers cytoprotective effects through the preservation of epithelial cell survival and homeostasis. Moreover, the anti-apoptotic activity of EMC may secondarily contribute to improved mucosal regeneration and barrier restoration, thereby mitigating the overall severity of chemotherapy-induced intestinal mucositis.

GSK-3β is a multifunctional serine/threonine kinase that operates as a downstream effector within the phosphoinositide 3-kinase (PI3K)/protein kinase B (AKT) signaling cascade and participates in multiple cellular pathways, including the Wnt/β-catenin axis [38]. The enzymatic activity of GSK-3β is tightly regulated by site-specific phosphorylation—phosphorylation at the Ser9 residue inhibits its kinase function, rendering p-GSK-3β the inactive form. Functionally, GSK-3β and cyclin D1 are both implicated in cell cycle progression, yet their interaction in cancer biology remains controversial. Several studies have demonstrated that active GSK-3β negatively regulates cyclin D1 expression in tumor cells, such as those derived from breast carcinoma and oral squamous carcinoma, suggesting a tumor-suppressive role. Conversely, other reports have found no clear association between GSK-3β activation and cyclin D1 expression in hepatocellular carcinoma or fibroblastic cells, implying a context-dependent regulatory mechanism [39]. In the present study, EMC treatment inhibited GSK-3β phosphorylation, thereby preventing the kinase from adopting an inactive conformation and enhancing its catalytic activity in pathways involved in inflammation, neurodegenerative diseases, and metabolic regulation [40]. This suggests that EMC may potentiate the antitumor efficacy of 5-FU through the modulation of GSK-3β activity, thereby promoting downstream effects associated with cell cycle arrest and apoptosis. Collectively, these findings highlight the pivotal role of GSK-3β/Cyclin D1 pathway in mediating the combined therapeutic response and underscore its potential as a molecular target in enhancing chemotherapeutic outcomes.

Intestinal mucositis induced by 5-FU involves severe epithelial disruption, which precipitates mucosal inflammation, barrier collapse, and inadequate epithelial repair. A key mechanism underlying this pathology is the induction of EMT, which drives the transition of intestinal epithelial cells toward a mesenchymal phenotype, facilitating loss of polarity and intercellular cohesion. The concomitant downregulation of β-catenin and E-cadherin further disrupts epithelial junctional complexes, heightening vulnerability to proinflammatory cytokines and pathogen penetration [40]. Conversely, the upregulation of N-cadherin, a mesenchymal marker protein, indicates a shift toward a mesenchymal phenotype, further aggravating intestinal mucosal damage. EMT contributes to intestinal inflammation through a reinforcing loop. Pro-inflammatory cytokines like TNF-α and IL-6 initiate EMT, which compromises barrier integrity. Inflammatory mediator release further aggravates mucosal inflammation, while EMT signaling orchestrates both epithelial injury and repair processes in 5-FU-induced intestinal mucositis [41]. By regulating key EMT markers—β-catenin, E-cadherin, and N-cadherin—tissue inflammation can be reduced and epithelial restoration enhanced. Our results demonstrate that EMC markedly inhibited 5-FU-induced EMT, evidenced by a downregulation of N-cadherin and upregulation of β-catenin and E-cadherin, thereby elucidating a mechanistic basis for its mucoprotective action.

Tight junction disruption is a pivotal pathological event in 5-FU-induced intestinal mucositis, as extensively characterized in preclinical murine models. These intercellular junctions are essential components of the intestinal epithelial barrier, composed primarily of structural and regulatory proteins such as occludin, claudins, and ZO-1 [42]. Together, these proteins maintain mucosal homeostasis by tightly controlling paracellular permeability and preventing luminal antigens, pathogens, and toxins from translocating into the underlying tissue. Following 5-FU administration, a pronounced decrease in ZO-1 expression was observed, indicative of compromised junctional organization and cytoskeletal anchorage, which subsequently led to elevated intestinal permeability. Similarly, occludin expression—a critical determinant of tight junction integrity and transmembrane barrier function—was markedly reduced in response to 5-FU exposure. This downregulation disrupts epithelial cohesion and weakens the mucosal defense against luminal insults [43]. Collectively, these alterations reflect the severe impairment of epithelial barrier architecture caused by 5-FU cytotoxicity, which facilitates microbial translocation and amplifies mucosal inflammation. Restoration or preservation of tight junction protein expression, therefore, represents a crucial therapeutic target for mitigating chemotherapy-induced mucosal injury and maintaining intestinal integrity. Loss of Claudin-1, in conjunction with diminished occludin and ZO-1 expression, was found to compromise the intestinal epithelial barrier following 5-FU administration. The resulting permeability increase permits luminal and systemic inflammatory mediators to penetrate the mucosa, aggravating tissue injury [44]. This barrier dysfunction likely stems from oxidative stress-mediated damage and the action of cytokines such as TNF-α and IL-6, both of which disrupt junctional protein synthesis and localization. EMC treatment significantly alleviated these alterations, suggesting its efficacy in restoring epithelial integrity by modulating oxidative and inflammatory pathways.

EMC is characterized by a rich profile of active compounds, notably p-coumaric acid, rutin, and quercetin, which collectively contribute to its diverse biological activities [45]. Previous studies have demonstrated that ethanol extracts of MC effectively ameliorate colon histopathological damage in experimental models of inflammatory bowel disease (IBD), underscoring the therapeutic relevance of targeting inflammation-associated cellular populations [46]. Building on previous work, which characterized EMC through HPLC–PAD fingerprinting and correlated its markers with pharmacological effects, EMC and its bioactive compounds (p-coumaric acid, rutin, and quercetin) may disrupt SARS-CoV-2 host cell entry by interfering with the ACE2/TMPRSS2 axis. This suggests a potential mechanism for their use as complementary natural agents against COVID-19 [17]. Furthermore, prophylactic administration of MC polysaccharides (MCP) was shown to attenuate ethanol-induced gastric mucosal injury in rats through the reduction in oxidative stress and inflammatory responses, primarily via inhibition of the NF-κB signaling pathway [47]. These findings collectively highlight the potential of MC bioactive constituents as multifunctional modulators of inflammation and oxidative damage, supporting their prospective application in gastrointestinal disorders such as mucositis.

P-coumaric acid, a naturally occurring phenolic compound, has shown promise in mitigating chemotherapy-induced mucositis through a multi-targeted mechanism of action relevant to mucosal injury and repair [48]. Its efficacy is largely attributed to the attenuation of two core pathological drivers: oxidative stress and inflammation. By directly scavenging chemotherapy-generated ROS, p-coumaric acid alleviates the initial oxidative insult to epithelial cells. Concurrently, it reduces the pivotal NF-κB signaling pathway, thereby downregulating the production of key pro-inflammatory cytokines (e.g., TNF-α, IL-6, IL-1β) and curtailing the inflammatory cascade [49]. Beyond this, p-coumaric acid exhibits direct cytoprotective functions within the mucosal context. It modulates the expression of Bcl-2 family proteins to inhibit the mitochondrial apoptotic pathway, promoting the survival of epithelial cells. Furthermore, its documented antimicrobial activity against common pathogens may help prevent secondary infections in the compromised mucosal barrier—a frequent complication that exacerbates mucositis and delays healing [50]. Collectively, by concurrently targeting oxidative damage, inflammatory signaling, apoptosis, and microbial challenge, p-coumaric acid addresses the multifaceted pathophysiology of mucositis, positioning it as a rational candidate for adjunctive therapy aimed at preserving mucosal integrity during chemotherapy [51].

Rutin, a flavonoid glycoside, demonstrates protective efficacy in models of chemotherapy-induced mucositis by concurrently targeting its core pathophysiological pathways [52]. The compound’s primary mechanism involves quenching chemotherapy-generated ROS and bolstering cellular antioxidant defenses, thereby mitigating the initial oxidative damage to the mucosal epithelium [53]. Furthermore, rutin potently reduces the inflammatory cascade central to mucositis progression. It achieves this primarily by inhibiting the activation and nuclear translocation of the transcription factor NF-κB, leading to reduced expression of key pro-inflammatory cytokines such as TNF-α and IL-6. At the cellular level, rutin promotes epithelial survival by modulating apoptotic regulators, shifting the balance toward anti-apoptotic signals [54]. This action helps preserve the integrity of the mucosal barrier. Importantly, emerging evidence suggests rutin may also actively support the healing phase, potentially by enhancing epithelial restitution through growth factor signaling pathways [55]. In summary, rutin offers a multi-mechanistic profile—encompassing antioxidant, anti-inflammatory, and cytoprotective activities—that aligns closely with the complex etiology of mucositis, positioning it as a viable candidate for adjunctive protective therapy.

Quercetin demonstrates significant protective potential against chemotherapy-induced mucositis by simultaneously targeting multiple, interconnected pathological mechanisms. Its efficacy stems from a capacity to mitigate the core drivers of mucosal injury [56]. Quercetin robustly counteracts oxidative stress by scavenging free radicals and enhancing endogenous cellular defenses via activation of the Nrf2/ARE pathway. In parallel, it reduces the predominant inflammatory cascade by inhibiting the NF-κB and MAPK signaling pathways, thereby reducing the production of key cytokines such as TNF-α and IL-6. At the cellular level, quercetin promotes epithelial survival by inhibiting the mitochondrial apoptotic pathway and helps maintain intestinal barrier integrity by supporting the expression of tight junction proteins like occludin and ZO-1 [57]. Notably, evidence suggests its protective effects may be amplified by prophylactic administration, which primes cellular defense systems before chemotherapy exposure. In summary, through its integrated modulation of oxidative, inflammatory, apoptotic, and barrier-disruptive processes, quercetin presents a multi-targeted therapeutic strategy for alleviating mucositis and supporting mucosal recovery during cytotoxic treatment [58].

Epicatechin, a key phenolic constituent of Momordica charantia pulp, works synergistically with compounds such as gallic acid, catechins, and chlorogenic acid to confer the fruit’s rich antioxidant profile [45]. While direct studies on epicatechin in the context of 5-FU-induced enteromucositis remain to be conducted, a growing body of indirect evidence strongly suggests its therapeutic promise. Therefore, findings from a 2018 study on epigallocatechin-3-gallate (EGCG) are particularly instructive: mice receiving 5-FU combined with EGCG exhibited markedly reduced mucositis severity and improved villus preservation compared to those treated with 5-FU alone. Given the structural and functional similarities between EGCG and epicatechin, it is reasonable to hypothesize that epicatechin may confer analogous protective effects [59]. Importantly, epicatechin has demonstrated efficacy in other models of mucosal injury. For example, in radiation-induced intestinal damage, epicatechin mitigated oxidative stress, decreased crypt cell apoptosis, and facilitated tissue regeneration via activation of the Nrf2 and Wnt/β-catenin pathways [60]. Mechanistically, epicatechin has been shown to interfere with critical inflammatory and oxidative processes central to mucositis, including the upregulation of NADPH oxidases (NOX1/NOX4), protein oxidation, and the activation of redox-sensitive NF-κB and ERK1/2 signaling in models of barrier disruption induced by high-fat diet or TNF-α [61]. Consequently, the accumulated evidence demonstrates that epicatechin enhances tight junction integrity, reduces inflammation, counteracts oxidative stress, and inhibits apoptosis—all of which directly target the pathological hallmarks of 5-FU-induced intestinal mucositis. Thus, dedicated studies employing specific 5-FU models are urgently needed to validate its therapeutic potential.

Synergistic effects have been observed when MC is combined with certain chemotherapeutic drugs. Specifically, it has been demonstrated that bitter melon extract (BME) inhibits ovarian cancer cell growth and overcomes cisplatin resistance through the activation of the AMPK signaling pathway [62]. Consequently, BME has been proposed as a potential supplement to improve the outcomes of cisplatin-based chemotherapy. Furthermore, MAP30, an active protein derived from bitter melon seeds, has been shown to possess potent anticancer and chemosensitizing properties. When administered in combination with cisplatin, a synergistic enhancement of cytotoxicity has been reported, and in animal studies, tumor spread and growth were significantly inhibited by low-dose combined injections without any observed impairment of liver or kidney function [63]. However, a contrasting finding has been reported in a clinical case, where acute pancreatitis was induced in a renal cancer patient receiving pazopanib following the consumption of bitter melon juice (approximately 100–150 mL daily for four days). The pancreatitis did not recur after pazopanib was resumed without bitter melon, and it was assessed that an interaction between bitter melon and pazopanib was the likely cause [64]. However, the effects of EMC are not uniform across all chemotherapeutic agents but are instead highly context-dependent, influenced by factors such as drug chemical structure, metabolic pathways, and therapeutic indices. Looking ahead, systematic drug interaction screening is urgently needed. Using standardized in vitro models, EMC and its various bioactive components should be evaluated against a broad panel of chemotherapeutic drugs representing different classes—including antimetabolites such as 5-fluorouracil, alkylating agents, taxanes, and targeted therapies—to assess both pharmacokinetic parameters and pharmacodynamic outcomes. Second, mechanistic studies are warranted to elucidate the effects of EMC on specific drug-metabolizing enzymes and transporters, thereby enabling the prediction of potential interactions before they are observed clinically. Third, future research must evaluate the impact of EMC on the efficacy of 5-FU in appropriate tumor-bearing animal models [45]. In these models, the protective effects of EMC on the intestinal mucosa and its antitumor activity against cancer cells can be monitored simultaneously, allowing for the identification of a therapeutic window in which EMC mitigates chemotherapy-induced toxicity without compromising the desired cytotoxic effects.

Overall, EMC is characterized by a diverse profile of bioactive polyphenolic compounds, notably *p*-coumaric acid, rutin, and quercetin. The synergistic interplay among these constituents forms a multifactorial therapeutic framework against mucosal ulceration. Collectively, these phytochemicals exert potent antioxidant and anti-inflammatory effects that directly counteract the core pathophysiological processes of mucositis. Specifically, they efficiently scavenge excessive ROS generated during chemotherapy, thereby preserving cellular integrity and mitigating oxidative tissue injury. Concurrently, inhibition of the NF-κB signaling cascade leads to marked downregulation of key pro-inflammatory mediators, including TNF-α and IL-6, thus attenuating mucosal inflammation. Moreover, modulation of Bcl-2 family proteins contributes to the reduction in aberrant apoptosis in epithelial cells, preserving mucosal barrier architecture and reducing ulcer formation. Taken together, these results underscore the synergistic cytoprotective potential of *p*-coumaric acid, rutin, and quercetin in mitigating mucositis through integrated antioxidant, anti-inflammatory, and anti-apoptotic mechanisms. Future mechanistic and translational studies are warranted to validate their combined efficacy and explore the clinical applicability of MC–derived polyphenols as adjunctive therapeutics for mucosal injury management.

## 5. Conclusions

In summary, the current study demonstrates that EMC effectively counteracts oxidative stress and histopathological deterioration induced by 5-FU in the small intestine. Through its potent antioxidant and anti-inflammatory actions, EMC reduces key pathological events—including inflammation, apoptosis, EMT, and barrier dysfunction—that contribute to mucositis progression. These findings position EMC as a potential candidate for adjunctive therapy in chemotherapy-induced intestinal toxicity. Future studies should explore its pharmacodynamic properties and long-term efficacy to establish a more complete understanding of its protective mechanisms.

## Data Availability

The original contributions presented in this study are included in the article. Further inquiries can be directed to the corresponding author. Sample Availability—Samples of the crude extracts prepared from *M. charantia* L. are available from the authors.

## Abbreviations

5-FU	5-Fluorouracil
EMC	The ethanolic extract of *M. charantia* L.
ROS	Reactive oxygen species
TBARS	Thiobarbituric acid reactive substance
MDA	Malondialdehyde
TBA	Thiobarbituric acid
DTNB	5,5′-dithiobis-(2-nitrobenzoic acid)
NO	Nitric oxide
TNF-α	Tumor necrosis factor-α
IL-1β	Interleukin-1β
IL-6	Interleukin-6
GSH	Glutathione
Inos	Inducible nitric oxide synthase
COX-2	Cyclooxygenase-2
TLRs	Toll-like receptors
TLR4	Toll-like receptor 4
MAPK	The mitogen-activated protein kinase
NF-κb	Nuclear Factor-κB
IKK	IκB kinase
IκB-α	NF-κB inhibitor α
SOD1	Superoxide dismutase 1
GPx3	Glutathione peroxidase 3
HO-1	Heme-oxygenase 1
Nrf2	Nuclear factor erythroid 2–related factor
GSK-3β	Glycogen synthase kinase-3β
ZO-1	Zonula occludens-1

## References

[1] Chukwunyere U., Mercan M., Sehirli A.O., Abacioglu N. (2022). Possible cytoprotective mechanisms of oxytocin against 5-fluorouracil-induced gastrointestinal mucositis. Mol. Biol. Rep..

[2] Voutsadakis I.A., Kokkali S., Digklia A. (2024). Treatment of metastatic biliary cancers with irinotecan and 5-Fluorouracil based chemotherapy after platinum/gemcitabine progression: A systematic review and meta-analysis. Clin. Color. Cancer.

[3] Alan N., Oran N.T., Yılmaz P.A., Çelik A., Yılmaz O. (2024). Fig seed oil improves intestinal damage caused by 5-FU-induced mucositis in rats. Food Sci. Nutr..

[4] Teng Y., Li J., Guo J., Yan C., Wang A., Xia X. (2024). Alginate oligosaccharide improves 5-fluorouracil-induced intestinal mucositis by enhancing intestinal barrier and modulating intestinal levels of butyrate and isovalerate. Int. J. Biol. Macromol..

[5] Ivanov S.M., Zgoda V.G., Isakova V.A., Trukhanova L.S., Poroikov V.V., Shtil A.A. (2024). Proteomic analysis identifies multiple mechanisms of 5-fluorouracil-induced gut mucositis in mice. Cancers.

[6] López-Gómez L., Alcorta A., Abalo R. (2023). Probiotics and probiotic-like agents against chemotherapy-induced intestinal mucositis: A narrative review. J. Pers. Med..

[7] Wang L., Song B., Hu Y., Chen J., Zhang S., Chen D., Wang J. (2021). Puerarin Ameliorates 5-Fluorouracil-Induced Intestinal Mucositis in Mice by Inhibiting JAKs. J. Pharmacol. Exp. Ther..

[8] Sethy C., Kundu C.N. (2021). 5-Fluorouracil (5-FU) resistance and the new strategy to enhance the sensitivity against cancer: Implication of DNA repair inhibition. Biomed. Pharmacother..

[9] Wei L., Wen X.S., Xian C.J. (2021). Chemotherapy-induced intestinal microbiota dysbiosis impairs mucosal homeostasis by modulating toll-like receptor signaling pathways. Int. J. Mol. Sci..

[10] Yoneda J., Nishikawa S., Kurihara S. (2021). Oral administration of cystine and theanine attenuates 5-fluorouracil-induced intestinal mucositis and diarrhea by suppressing both glutathione level decrease and ROS production in the small intestine of mucositis mouse model. BMC Cancer.

[11] Alcorta A., López-Gómez L., Capasso R., Abalo R. (2024). Vitamins and fatty acids against chemotherapy-induced intestinal mucositis. Pharmacol. Ther..

[12] Blondy S., David V., Verdier M., Mathonnet M., Perraud A., Christou N. (2020). 5-Fluorouracil resistance mechanisms in colorectal cancer: From classical pathways to promising processes. Cancer Sci..

[13] Ghafouri-Fard S., Abak A., Tondro Anamag F., Shoorei H., Fattahi F., Javadinia S.A., Basiri A., Taheri M. (2021). 5-Fluorouracil: A narrative review on the role of regulatory mechanisms in driving resistance to this chemotherapeutic agent. Front. Oncol..

[14] Xu B., Li Z., Zeng T., Zhan J., Wang S., Ho C.T., Li S. (2022). Bioactives of *Momordica charantia* as Potential Anti-Diabetic/Hypoglycemic Agents. Molecules.

[15] Richter E., Geetha T., Burnett D., Broderick T.L., Babu J.R. (2023). The Effects of *Momordica charantia* on Type 2 Diabetes Mellitus and Alzheimer’s Disease. Int. J. Mol. Sci..

[16] Kao P.F., Cheng C.H., Cheng T.H., Liu J.C., Sung L.C. (2024). Therapeutic Potential of Momordicine I from *Momordica charantia*: Cardiovascular Benefits and Mechanisms. Int. J. Mol. Sci..

[17] Chao C.Y., Shyu W.C., Lin C.L., Jiang W.P., Inose A., Chiang S.J., Wu W.L., Lin J.G., Huang G.J. (2026). Bioactive Compounds of *Momordica charantia* L. Downregulate the Protein Expression of ACE2 and TMPRSS2 In Vivo and In Vitro. Int. J. Mol. Sci..

[18] Lin C.H., Jiang W.P., Itokazu N., Huang G.J. (2025). Chlorogenic acid attenuates 5-fluorouracil-induced intestinal mucositis in mice through SIRT1 signaling-mediated oxidative stress and inflammatory pathways. Biomed. Pharmacother..

[19] Lin J.G., Sun Y.W., Wu W.L., Jiang W.P., Zhung F.Y., Huang G.J. (2025). Multi-Target Protective Effects of *Sanghuangporus sanghuang* Against 5-Fluorouracil-Induced Intestinal Injury Through Suppression of Inflammation, Oxidative Stress, Epithelial-Mesenchymal Transition, and Tight Junction. Int. J. Mol. Sci..

[20] Kim D.R., Kim J., Oh J.Y., Kim H.Y., Kim Y.J., Chang M.S. (2017). Protective effect of *Salvia miltiorrhiza* Bunge on 5-fluorouracil-induced oral mucositis. Int. J. Mol. Med..

[21] Likitsatian T., Koonyosying P., Paradee N., Roytrakul S., Ge H., Pourzand C., Srichairatanakool S. (2025). Camellia Tea Saponin Ameliorates 5-Fluorouracil-Induced Damage of HaCaT Cells by Regulating Ferroptosis and Inflammation. Nutrients.

[22] Yin Q., Li X., Xiong Y., Jiang Y., Ma S., Qian G. (2025). Bletilla oligosaccharides improved 5-fluorouracil-induced intestinal mucositis in mice by activating NF-kappaB signalling pathway and regulating intestinal microbiota. Front. Pharmacol..

[23] Chien L.H., Deng J.S., Jiang W.P., Chou Y.N., Lin J.G., Huang G.J. (2023). Evaluation of lung protection of *Sanghuangporus sanghuang* through TLR4/NF-kappaB/MAPK, keap1/Nrf2/HO-1, CaMKK/AMPK/Sirt1, and TGF-beta/SMAD3 signaling pathways mediating apoptosis and autophagy. Biomed. Pharmacother..

[24] Atiq A., Shal B., Naveed M., Khan A., Ali J., Zeeshan S., Al-Sharari S.D., Kim Y.S., Khan S. (2019). Diadzein ameliorates 5-fluorouracil-induced intestinal mucositis by suppressing oxidative stress and inflammatory mediators in rodents. Eur. J. Pharmacol..

[25] Chien L.H., Wu C.T., Deng J.S., Jiang W.P., Huang W.C., Huang G.J. (2021). Salvianolic Acid C protects against cisplatin-induced acute kidney injury through attenuation of inflammation, oxidative stress and apoptotic effects and activation of the CaMKK-AMPK-Sirt1-associated signaling pathway in mouse models. Antioxidants.

[26] Lin W.H., Jiang W.P., Chen C.C., Lee L.Y., Tsai Y.S., Chien L.H., Chou Y.N., Deng J.S., Huang G.J. (2022). Renoprotective effect of *Pediococcus acidilactici* GKA4 on cisplatin-induced acute kidney injury by mitigating inflammation and oxidative stress and regulating the MAPK, AMPK/SIRT1/NF-κB, and PI3K/AKT pathways. Nutrients.

[27] Cheng M.R., Li Q., Wan T., He B., Han J., Chen H.X., Yang F.X., Wang W., Xu H.Z., Ye T. (2012). Galactosylated chitosan/5-fluorouracil nanoparticles inhibit mouse hepatic cancer growth and its side effects. World J. Gastroenterol..

[28] Al-Hoshary D.M., Zalzala M.H. (2023). Mucoprotective effect of ellagic acid in 5 fluorouracil-induced intestinal mucositis model. J. Med. Life.

[29] Jiang W.P., Deng J.S., Huang S.S., Wu S.H., Chen C.C., Liao J.C., Chen H.Y., Lin H.Y., Huang G.J. (2021). *Sanghuangporus sanghuang* mycelium prevents paracetamol-induced hepatotoxicity through Regulating the MAPK/NF-kappaB, Keap1/Nrf2/HO-1, TLR4/PI3K/Akt, and CaMKKbeta/LKB1/AMPK pathways and suppressing oxidative stress and inflammation. Antioxidants.

[30] Lin W.C., Deng J.S., Huang S.S., Wu S.H., Chen C.C., Lin W.R., Lin H.Y., Huang G.J. (2017). Anti-inflammatory activity of *Sanghuangporus sanghuang* mycelium. Int. J. Mol. Sci..

[31] Li T., Si W., Zhu J., Yin L., Zhong C. (2020). Emodin reverses 5-Fu resistance in human colorectal cancer via downregulation of PI3K/Akt signaling pathway. Am. J. Transl. Res..

[32] Huang J., Hwang A.Y.M., Jia Y., Kim B., Iskandar M., Mohammed A.I., Cirillo N. (2022). Experimental chemotherapy-induced mucositis: A scoping review guiding the design of suitable preclinical models. Int. J. Mol. Sci..

[33] Hassanein E.H.M., Althagafy H.S., Mansour S.M.A., Omar Z.M.M., Hussein Hassanein M.M., Abd El-Ghafar O.A.M. (2024). Vinpocetine attenuates 5-fluorouracil-induced intestinal injury: Role of the Keap1/Nrf2/HO-1, NF-kappaB/TLR4/SOCS3 and RIPK1/RIPK3/MLKL signals. Immunopharmacol. Immunotoxicol..

[34] Ali J., Khan A.U., Shah F.A., Ali H., Islam S.U., Kim Y.S., Khan S. (2019). Mucoprotective effects of Saikosaponin-A in 5-fluorouracil-induced intestinal mucositis in mice model. Life Sci..

[35] Kim H.J., Kim J.H., Moon W., Park J., Park S.J., Song G.A., Han S.H., Lee J.H. (2015). Rebamipide attenuates 5-Fluorouracil-induced small intestinal mucositis in a mouse model. Biol. Pharm. Bull..

[36] Xu L., Zhao X., Tang F., Zhang J., Peng C., Ao H. (2024). Ameliorative Effect of Ginsenoside Rc on 5-Fluorouracil-Induced Chemotherapeutic Intestinal Mucositis via the PI3K-AKT/NF-kappaB Signaling Pathway: In Vivo and In Vitro Evaluations. Int. J. Mol. Sci..

[37] Khan S., Wardill H.R., Bowen J.M. (2018). Role of toll-like receptor 4 (TLR4)-mediated interleukin-6 (IL-6) production in chemotherapy-induced mucositis. Cancer Chemother. Pharmacol..

[38] Ji L., Hao S., Wang J., Zou J., Wang Y. (2022). Roles of toll-like receptors in radiotherapy- and chemotherapy-induced oral mucositis: A concise review. Front. Cell. Infect. Microbiol..

[39] Liao Y.F., Luo F.L., Tang S.S., Huang J.W., Yang Y., Wang S., Jiang T.Y., Man Q., Liu S., Wu Y.Y. (2022). Network analysis and experimental pharmacology study explore the protective effects of Isoliquiritigenin on 5-fluorouracil-induced intestinal mucositis. Front. Pharmacol..

[40] Xie M., Lin W., Du Y., Li Y., Li S. (2025). AHNAK2 confers 5-fluorouracil resistance in colorectal cancer via activation of the AKT/GSK-3beta signaling axis. Clin. Exp. Med..

[41] Niu Y., Xiao H., Wang B., Wang Z., Du K., Wang Y., Wang L. (2023). Angelica sinensis polysaccharides alleviate the oxidative burden on hematopoietic cells by restoring 5-fluorouracil-induced oxidative damage in perivascular mesenchymal progenitor cells. Pharm. Biol..

[42] Manfioletti G., Fedele M. (2023). Epithelial-Mesenchymal Transition (EMT). Int. J. Mol. Sci..

[43] Chattopadhyay I., Ambati R., Gundamaraju R. (2021). Exploring the crosstalk between inflammation and epithelial-mesenchymal transition in cancer. Mediat. Inflamm..

[44] Kaminsky L.W., Al-Sadi R., Ma T.Y. (2021). IL-1beta and the intestinal epithelial tight junction barrier. Front. Immunol..

[45] Cuong D.M., Kwon S.J., Jeon J., Park Y.J., Park J.S., Park S.U. (2018). Identification and Characterization of Phenylpropanoid Biosynthetic Genes and Their Accumulation in Bitter Melon (*Momordica charantia*). Molecules.

[46] Semiz A., Ozgun Acar O., Cetin H., Semiz G., Sen A. (2020). Suppression of Inflammatory Cytokines Expression with Bitter Melon (*Momordica charantia*) in TNBS-instigated Ulcerative Colitis. J. Transl. Int. Med..

[47] Raish M., Ahmad A., Ansari M.A., Alkharfy K.M., Aljenoobi F.I., Jan B.L., Al-Mohizea A.M., Khan A., Ali N. (2018). *Momordica charantia* polysaccharides ameliorate oxidative stress, inflammation, and apoptosis in ethanol-induced gastritis in mucosa through NF-kB signaling pathway inhibition. Int. J. Biol. Macromol..

[48] Chen F., Zhang X., Wang J., Wang F., Mao J. (2024). P-coumaric Acid: Advances in Pharmacological Research Based on Oxidative Stress. Curr. Top. Med. Chem..

[49] Yoon J.H., Youn K., Ho C.T., Karwe M.V., Jeong W.S., Jun M. (2014). *p*-Coumaric acid and ursolic acid from *Corni fructus* attenuated beta-amyloid_25-35_-induced toxicity through regulation of the NF-kappaB signaling pathway in PC12 cells. J. Agric. Food Chem..

[50] Kheiry M., Dianat M., Badavi M., Mard S.A., Bayati V. (2019). p-Coumaric Acid Attenuates Lipopolysaccharide-Induced Lung Inflammation in Rats by Scavenging ROS Production: An In Vivo and In Vitro Study. Inflammation.

[51] Kincses A., Ghazal T.S.A., Hohmann J. (2024). Synergistic effect of phenylpropanoids and flavonoids with antibiotics against Gram-positive and Gram-negative bacterial strains. Pharm. Biol..

[52] Pandey P., Khan F., Qari H.A., Oves M. (2021). Rutin (Bioflavonoid) as Cell Signaling Pathway Modulator: Prospects in Treatment and Chemoprevention. Pharmaceuticals.

[53] Muvhulawa N., Dludla P.V., Ziqubu K., Mthembu S.X.H., Mthiyane F., Nkambule B.B., Mazibuko-Mbeje S.E. (2022). Rutin ameliorates inflammation and improves metabolic function: A comprehensive analysis of scientific literature. Pharmacol. Res..

[54] Imani A., Maleki N., Bohlouli S., Kouhsoltani M., Sharifi S., Maleki Dizaj S. (2021). Molecular mechanisms of anticancer effect of rutin. Phytother. Res..

[55] Wu L., Li L., Wang X., Wu H., Li M., Wang Y., Sheng P., An X., Yan M. (2024). The inhibition of rutin on Src kinase blocks high glucose-induced EGFR/ERK transactivation in diabetic nephropathy by integrative approach of network pharmacology and experimental verification. Phytomedicine.

[56] Qi W., Qi W., Xiong D., Long M. (2022). Quercetin: Its Antioxidant Mechanism, Antibacterial Properties and Potential Application in Prevention and Control of Toxipathy. Molecules.

[57] Alharbi H.O.A., Alshebremi M., Babiker A.Y., Rahmani A.H. (2025). The Role of Quercetin, a Flavonoid in the Management of Pathogenesis Through Regulation of Oxidative Stress, Inflammation, and Biological Activities. Biomolecules.

[58] Lei P. (2025). Potential Roles of Exercise and Quercetin in Modulating Cancer Pathways and Cognitive Function. Phytother. Res..

[59] Vladu A.F., Ficai D., Ene A.G., Ficai A. (2022). Combination therapy using polyphenols: An efficient way to improve antitumoral activity and reduce resistance. Int. J. Mol. Sci..

[60] Takahashi N., Kobayashi M., Ogura J., Yamaguchi H., Satoh T., Watanabe K., Iseki K. (2014). Immunoprotective effect of epigallocatechin-3-gallate on oral anticancer drug-induced α-defensin reduction in Caco-2 cells. Biol. Pharm. Bull..

[61] Li Y., Cheng L., Li M. (2024). Effects of green tea extract epigallocatechin-3-gallate on oral diseases: A narrative review. Pathogens.

[62] Deligiannidou G.E., Pritsa A., Nikolaou A., Poulios E., Kontogiorgis C., Papadopoulou S.K., Giaginis C. (2025). Nutraceutical potential of bitter melon (*Momordica charantia*) in cancer treatment: An overview of in vitro and animal studies. Curr. Issues Mol. Biol..

[63] Chan D.W., Yung M.M., Chan Y.S., Xuan Y., Yang H., Xu D., Zhan J.B., Chan K.K., Ng T.B., Ngan H.Y. (2020). MAP30 protein from *Momordica charantia* is therapeutic and has synergic activity with cisplatin against ovarian cancer in vivo by altering metabolism and inducing ferroptosis. Pharmacol. Res..

[64] Unsal O., Sütcüoğlu O., Yazıcı O. (2022). Dangerous interaction of bitter melon (*Momordica charantia*) with pazopanib: A case of acute pancreatitis. J. Oncol. Pharm. Pract..

